# Identification of a shared antigen linking CD4^+^ T and B cell pathology in Sjögren’s disease

**DOI:** 10.1126/sciadv.aeb2491

**Published:** 2026-06-03

**Authors:** Masaru Takeshita, Jun Inamo, Seiki Wakui, Ryosuke Nagashima, Takahiro Nishino, Kazuyuki Tsunoda, Satoshi Usuda, Hajime Inokuchi, Kazuyoshi Ishigaki, Takashi Sasaki, Yuki Kagoya, Katsuya Suzuki, Yuko Kaneko

**Affiliations:** ^1^ Division of Rheumatology, Department of Internal Medicine, Keio University School of Medicine, 35 Shinanomachi, Shinjuku-ku, Tokyo 160-8582, Japan.; ^2^ Division of Rheumatology, University of Colorado School of Medicine, Aurora, CO 80045, USA.; ^3^ Department of Microbiology and Immunology, Keio University School of Medicine, Tokyo, Japan.; ^4^ Laboratory for Human Immunogenetics, RIKEN Center for Integrative Medical Sciences, Yokohama, Japan.; ^5^ JSR-Keio University Medical and Chemical Innovation Center (JKiC), JSR Corp., 35 Shinanomachi, Shinjuku-ku, Tokyo 160-8582, Japan.; ^6^ Department of Dentistry and Oral Surgery, Keio University School of Medicine, Shinjuku-ku, Tokyo 160-8582, Japan.; ^7^ Keio University Human Biology-Microbiome-Quantum Research Center (WPI-Bio2Q), Shinjuku-ku, Tokyo, 160-8582, Japan.; ^8^ Center for Supercentenarian Medical Research, Keio University School of Medicine, 35 Shinanomachi, Shinjuku-ku, Tokyo 160-8582, Japan.; ^9^ Division of Tumor Immunology, Institute for Advanced Medical Research, Keio University School of Medicine, 35 Shinanomachi, Shinjuku-ku, Tokyo 160-8582, Japan.; ^10^ Division of Rheumatology, Department of Internal Medicine, NHO Tokyo Medical Center, Meguro-ku, Tokyo 152-8902, Japan.

## Abstract

Sjögren’s disease (SjD) is an autoimmune disorder characterized by lymphocytic infiltration of exocrine glands. Although B cells producing anti-Ro60 autoantibodies are frequently found in salivary gland lesions, the antigen specificity of CD4^+^ T cells has remained unclear. Given accumulating evidence for T cell involvement in local autoantibody production, we comprehensively investigated Ro60 reactivity among lesion-infiltrating CD4^+^ T cells. Over 200 T cell receptors (TCRs) enriched in salivary glands from Japanese and Caucasian patients with SjD were screened using a TCR reporter system, identifying 13 Ro60-reactive TCRs predominantly expressed in T peripheral helper/T follicular helper subset, along with human leukocyte antigen alleles linked to SjD susceptibility. Ro60 was efficiently phagocytosed by antigen-presenting cells in the presence of autoantibodies and presented to Ro60-specific T cells, triggering their activation. This suggests that Ro60-specific B and CD4^+^ T cells orchestrate a pathological loop in salivary glands. Our study provides the first molecular identification of a major CD4^+^ T cell antigen in systemic autoimmunity and highlights coordinated T-B cell responses against a shared autoantigen.

## Teaser

Ro60-specific CD4^+^ T cells and B cells coordinate a pathological loop in the salivary glands of patients with Sjögren’s disease.

## INTRODUCTION

Sjögren’s disease (SjD) is a common autoimmune disease primarily characterized by chronic inflammation and destruction of the lacrimal and salivary glands. In some patients, the disease progresses beyond exocrine tissues to affect organs such as the lungs, kidneys, or peripheral nerves. Lymphocytic infiltration is a hallmark of the lesions, with early T cell predominance followed by B cell infiltration. In certain cases, these infiltrates form ectopic lymphoid structures, which contribute to chronic immune activation and, in some patients, hypergammaglobulinemia or B cell lymphoma (*1*, *2*). Serum autoantibodies are commonly detected: anti–Sjögren’s syndrome antigen A (Ro) (anti-SSA/Ro) in ~70%, anti–Sjögren’s syndrome antigen B (La) (anti-SSB/La) in ~30%, and anti-centromere antibodies in ~15%. Anti-SSA/Ro antibodies target two distinct proteins, Ro60 and Ro52; antibodies to Ro60 or both Ro60 and Ro52 are associated with SjD and systemic lupus erythematosus (SLE), while anti-Ro52 alone is found in a broader range of diseases (*3*). Notably, B cells producing anti-SSA/Ro and anti-SSB/La antibodies have long been recognized within salivary gland tissues (*4*, *5*).

To comprehensively characterize the lymphocytes infiltrating the lesion, we recently performed a comprehensive analysis of the antigen specificity of B cells. We generated over 250 monoclonal antibodies from salivary gland–infiltrating antibody-producing cells and revealed that ~30% of them were reactive to Ro60 and centromere antigens in patients with serum anti-SSA/Ro and anti-centromere antibodies, respectively. These antibodies have undergone affinity maturation and recognize various epitopes on the overall autoantigens, indicating that autoantibodies are actively produced at the lesion site in an antigen-driven manner (*6*–*8*). Similar patterns of autoantibody production have been observed at the lesion site of interstitial lung disease associated with rheumatoid arthritis (RA), SjD, mixed connective tissue disease (*9*), and anti-synthetase syndrome (*10*), suggesting that infiltrated B cells commonly target disease-specific autoantigens. However, the targets of T cells in these conditions remain largely unidentified.

CD4^+^ T cells also infiltrate the salivary glands and are critical in immune regulation. Despite the fact that class II human leukocyte antigen (HLA) alleles, which present antigenic peptides to CD4^+^ T cells, are the strongest genetic risk factors for SjD identified by genome-wide association studies (*11*), the antigenic peptides presented by these HLA molecules and recognized by CD4^+^ T cells remain largely unknown, due to the complexity of T cell receptor (TCR)–HLA/peptide recognition and the relatively low affinity of TCR interactions (*12*). Although several T cell antigens such as Ro52 (*13*), alpha amylase (*14*), and M3 muscarinic acetylcholine receptor (*15*) have been reported, findings have been limited and fragmented. Newer approaches such as TScan-II have identified candidates such as DDIAS and MAP3K4 (*16*), but with limited TCR coverage, leaving the major CD4^+^ T cell targets in SjD unclear.

Given our findings of active anti-Ro60 antibody production in the glands, and the enrichment of T follicular helper (T_FH_) and T peripheral helper (T_PH_) cells in both the peripheral blood and salivary glands of patients with SjD (*17*–*20*), we hypothesized that Ro60 might also serve as a major CD4^+^ T cell antigen in SjD. We compiled a library of over 200 clonally expanded TCRs from Ro60 antibody-positive lesions, expressed them in reporter cells, and assessed their reactivity against Ro60 peptides presented on matched HLA class II molecules. This approach enabled the identification of disease-driving TCRs and their corresponding HLA/peptide complexes, detailed in this study.

## RESULTS

### Identification of class II HLAs frequently found in Japanese patients with SjD

Previous HLA studies in SjD have shown that haplotypes including HLA-DRB1*03:01 in Caucasians and HLA-DRB1*08:03 and DRB1*04:05 in Asians are associated with anti-SSA/Ro antibody-positive SjD (*21*–*23*), although it was not clearly distinguished whether the antigen for anti-SSA/Ro antibody was Ro60 and Ro52. We first performed HLA typing of 35 patients with SjD (corresponding to 70 HLA class II alleles) to identify highly frequent HLAs in our target cohort of serum anti-Ro60 antibody-positive Japanese patients. As shown in Table 1, the haplotypes including DRB1*08:03, DRB1*15:02, and DRB1*15:01 accounted for approximately half of the allele frequencies and also had higher odds ratios (ORs) compared with controls. As previously reported (*24*), an intense linkage disequilibrium was observed between DR and DQ, and all but one DRs and DQs were associated. Among them, DRB1*08:03-DQA1*01:03-DQB1*06:01 and DRB1*15:01-DQA1*01:02-DQB1*06:02 were significantly enriched in serum anti-Ro60 antibody-positive Japanese patients with SjD. Although not statistically significant due to its low allele frequency, DRB1*14:06 exhibited the highest OR (3.337) among the tested alleles. DPB1*05:01 was observed in over half of the alleles from the disease group with an OR of 1.698, although not statistically significant [false discovery rate (FDR) = 0.102]. Based on these findings, we collected salivary gland samples from serum anti-Ro60 antibody-positive patients with SjD carrying these HLA alleles. The clinical information for patients is shown in table S1.

**Table 1.** Allele frequency in patients with SjD. Ab, antibody.

HLA type	Associated DQA1/DQB1	Allele frequency in serum anti-Ro60 Ab^+^ patients with SjD (%)	Allele frequency in Japanese database (%)	Odds ratio	*P* value^*^
DRB1		***n* = 70**	***n* = 48,946**		
08:03	DQA1^*^01:03/DQB1^*^06:01	17.1	8.2	2.320	0.014^†^
15:02	DQA1^*^01:03/DQB1^*^06:01	15.7	10.6	1.566	0.173
15:01	DQA1^*^01:02/DQB1^*^06:02	14.3	7.6	2.027	0.042^†^
09:01	DQA1^*^03/DQB1^*^03:03	11.4	14.7	0.751	0.611
04:05	DQA1^*^03/DQB1^*^04:01^‡^	7.1	13.1	0.510	0.158
14:06	DQA1^*^05:03/DQB1^*^03:01	4.3	1.3	3.337	0.067
13:02	DQA1^*^01:02/DQB1^*^06:04	4.3	6.1	0.695	0.443
04:06	DQA1^*^03/DQB1^*^03:02	4.3	3.3	1.326	0.500
**DPB1**		***n* = 70**	***n* = 2966**		
05:01		51.4	38.4	1.698	0.034
02:01		20.0	24.1	0.787	0.481
09:01		10.0	9.9	1.006	1.000
03:01		7.1	4.0	1.857	0.205

* Fisher’s exact test.

† FDR < 0.1 by Benjamini-Hochberg procedure.

‡ One patient did not have DQB1*04:01.

### Gene expression and TCR repertoire analysis of salivary glands of SjD

**Fig. 1.**
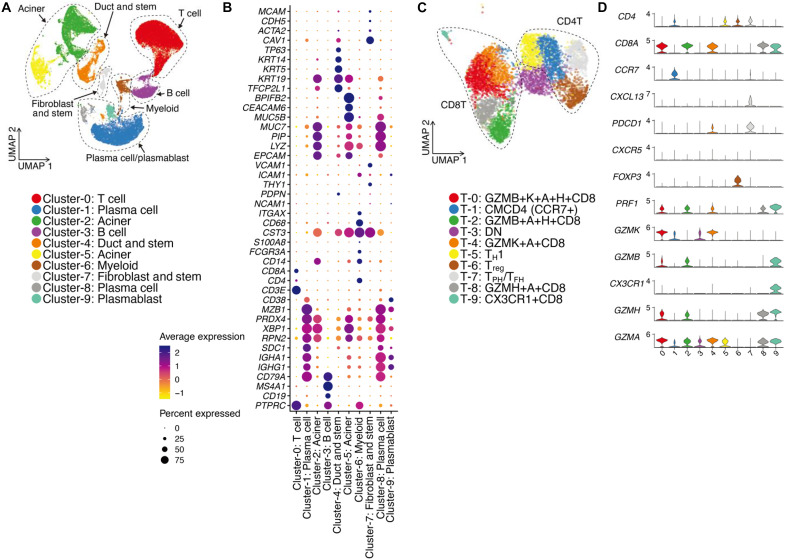
Identification of CD4^+^ T cells in the salivary glands of patients with SjD. (**A**) The uniform manifold approximation and projection (UMAP) plot displays broad cell types identified in the salivary glands. (**B**) Marker gene expressions in different cell types. (**C**) T cells were plotted in the UMAP plot. Four CD4^+^ T cell subclusters were identified. T_H_1, T helper 1. (**D**) Marker gene expression in different T cell subclusters is shown.

Next, we performed single-cell RNA sequencing (scRNA-seq) analysis and TCR repertoire analysis of salivary gland tissues from patients with SjD. In gene expression analysis, after correcting background noise and technical batch, we observed cell clusters including T cells, B cells, plasma cells, myeloid cells including macrophages, and epithelial cells (Fig. 1, A and B). Among them, we focused on the T cell cluster and further subdivided them into distinct subclusters based on the expression of marker genes (Fig. 1, C and D, and fig. S1A); among them, cluster 7 was considered to be T_PH_/T_FH_ cells because of the expression of *CD4*, *CXCL13*, *PDCD1*, *ICOS*, and *CXCR5.* Cluster 6 was considered as regulatory T cells (T_reg_ cells) because of the specific expression of *FOXP3* and *IL2RA*. The distribution of T cells in each sample is shown in fig. S1 (B and C), with slightly more CD8^+^ T cells in LB220, although no drastic bias was observed.

Next, to select the TCRs of CD4^+^ T cells enriched in the disease lesions, we listed sequences of TCRs common to at least three cells in LB219, which had a high number of cells, and at least two cells in the other samples. In addition, TCR sequences were analyzed by direct sorting of CD4^+^ T cells from the salivary glands of two patients, LB63 and LB65. From each of the 32 sorted cells, 25 and 20 TCRs each, for which both TCRα and β chain sequences could be identified, were also included in the analysis.

### Confirmation of local production of anti-Ro60 antibodies in the salivary glands

Since it was not clear whether anti-Ro60 antibodies were produced in the tissues of all serum anti-Ro60 antibody-positive patients with SjD, we further confirmed the production of anti-Ro60 antibodies in the tissues. From three patients, LB183, LB189, and LB216, we generated disease lesion-derived monoclonal antibodies in vitro, and it was confirmed that 9.5 to 27.5% of these antibodies could bind to Ro60 (fig. S2A). In six patients, LB183, LB189, LB215, LB219, LB220, and LB221, we performed immunostaining using fluorochrome-conjugated Ro60 and anti-CD138 antibodies; we confirmed the presence of anti-Ro60 antibody-producing cells in the salivary glands except for one case, LB220 (fig. S2B). LB183 and LB189 were confirmed in both methods, and a consistent trend was observed with LB189 producing more anti-Ro60 antibodies in the salivary glands than LB183. Last, we decided to proceed with the analysis of TCR specificity in eight samples (LB183, LB189, LB215, LB216, LB219, LB221, LB63, and LB65). The number of tested TCRs is included in table S1.

### Determination of antigen specificity of CD4^+^ T cells

**Fig. 2.**
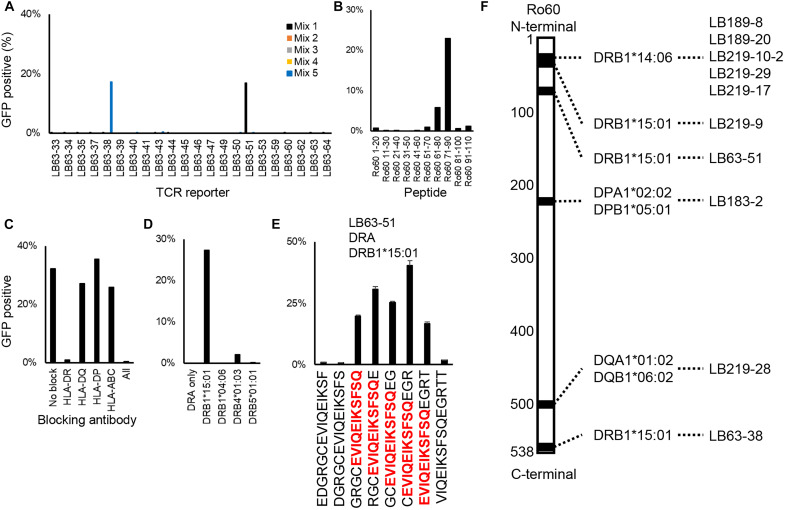
Identification of T cell epitopes. (**A**) The reporter cells were cocultured with LCLs from the same patient and a mixture of 10- of 20-mer peptides with a 10–amino acid overlap. GFP expression in the reporter cells was measured the following day. Representative results of the LB63-derived TCR reporters are shown. LB63-38 reacted with peptide mixture 5 and LB63-51 reacted with peptide mixture 1. (**B**) The LB63-51 reporter and LCLs were cocultured with each single peptide in peptide mixture 1. This reporter reacted with Ro60_71–90_. (**C**) The LB63-51 reporter and LCLs were cocultured with Ro60_71–90_ peptide after 1-hour incubation with blocking antibodies against HLA-DR, HLA-DQ, HLA-DP, and HLA-ABC. This reporter was blocked by anti-HLA-DR antibody. (**D**) The LB63-51 reporter was cocultured with 293T cells expressing one of the LB63 patient HLA-DR alleles with Ro60_71–90_ peptide. (**E**) LB63-51 reporter cells were cocultured with HLA-DR*15:01 expressing 293T and serial overlapping 15-mer peptides covering the 66 to 87 region of Ro60. The core epitopes recognized by LB63-51 are shown in red. Data are shown as the median and SD of duplicates. (**F**) The distribution of T cell epitopes in the Ro60 and the corresponding HLA and TCR are shown. The black areas indicate T cell epitopes.

Next, we expressed the listed TCRs on TCR reporter cells using retroviruses to generate a TCR reporter library consisting of 176 TCRs. To determine the antigen specificity of these reporters, we performed a stepwise epitope determination strategy as shown in a representative example in Fig. 2. We prepared 20-mer peptides with a 10–amino acid overlap over the entire Ro60 length of 538 amino acids and then created a mixture of ~10 of these peptides together. First, reporter cells and lymphoblastoid cell lines (LCLs) from the same patient were cocultured with one peptide mixture per well (Fig. 2A). Reporters that responded to any of the mixtures were then cocultured with LCLs and one peptide in the mixture per well to identify potential epitope sites roughly (Fig. 2B). Next, to determine the corresponding HLA types, reporters were cocultured with reacted peptide and LCLs after treatment with blocking antibodies against each HLA type (Fig. 2C). The response was usually inhibited by one of the blocking antibodies. We then used 293T cells expressing the individual HLA alleles that the patient had and identified the corresponding alleles (Fig. 2D). After determining the HLA alleles and rough epitope sites, last, we identified the core sequence using serial overlapping 15-mer peptides covering around the rough epitope site (Fig. 2E).

In total, 10 Ro60-specific TCRs were identified, and their core epitopes and corresponding HLA alleles are summarized in Fig. 2F and fig. S3 (A to E). Epitopes were distributed throughout the Ro60 protein, and five different TCRs from two patients reacted to the same epitope presented by DRB1*14:06, a rare allele with a high OR. In addition, we identified three TCRs that reacted to DRB1*15:01, which were enriched in anti-Ro60 antibody-positive patients (OR = 2.027), one TCR that reacted to DQA1*01:02/DQB1*06:02, which is in intense linkage disequilibrium with DRB1*15:01, and one TCR that reacted to DPB1*05:01 with an OR of 1.698, although not statistically significant.

The groove of the class II HLA is approximately nine amino acids in length, and the average length of the peptide presented is ~15 amino acids. When we tried shorter sequences, we found that the TCR reacted efficiently with an 11–amino acid peptide, approximately one amino acid on each side of the core sequence (fig. S3F). Together, multiple TCRs were found to be specific for Ro60 in multiple patients, providing confirmation of our hypothesis that there are CD4^+^ T cells corresponding to anti-Ro60 antibody-producing cells in the salivary glands.

To ensure that our findings were not driven by nonspecific activation, we performed two negative control experiments. First, we tested whether TCR reporters derived from anti-Ro60–positive patients responded to myeloperoxidase-derived peptide pools, an autoantigen unrelated to SjD (fig. S4, A and B). Second, we evaluated whether TCRs derived from our previously published dataset (*25*) from patients who were seropositive for anti-centromere antibodies but seronegative for anti-Ro60 antibodies reacted to Ro60 peptide pools (fig. S4C). In both experiments, no reporter activation was detected, confirming that Ro60-specific T cells are present only in individuals who are seropositive for anti-Ro60 antibodies.

### Validation of identified TCR epitopes

We identified the epitope of the TCR using synthetic peptides, but Ro60 is expressed as a full-length protein in vivo and is not present in the peptide form. Therefore, we examined whether the identified epitopes could indeed be presented on HLAs in two different ways.

**Fig. 3.**
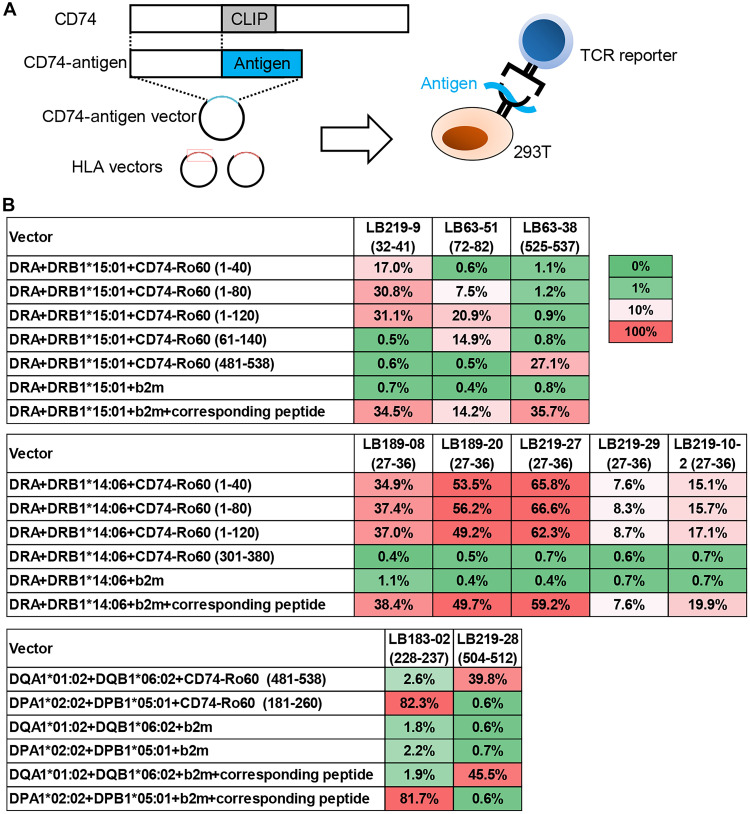
Validation of identified epitopes using expression vectors. (**A**) Schema of antigen presentation experiments using CD74-antigen fusion protein–expressing vectors. CD74-antigen vectors were generated by replacing the sequence behind from CLIP of CD74 with the antigen sequence. The fusion vector and HLA vectors were cotransfected into 293T cells to present the HLA/peptide complex. (**B**) The proportion of GFP-positive cells among reporter cells after overnight coculture with 293T cells transfected with the indicated vectors. The numbers after Ro60 indicate the position of the amino acid used as an antigen. The numbers under TCR reporter indicate the core epitopes. A β2 microglobulin (b2m)–expressing vector was used as the negative control.

The first experiment was performed using expression vectors. When class II HLA is synthesized, an amino acid sequence called CLIP in the CD74 molecule binds to the groove of the HLA molecule to prevent unwanted peptides from binding to the groove. It has been shown that this mechanism can be used to present antigens on HLA by replacing the post-CLIP portion of the CD74 sequence with a sequence of up to ~100 amino acids including the sequence of the antigen peptide (*16*, *26*). Using this mechanism, we recombined the post-CLIP portion into nucleic acid sequences coding for 40, 80, and 120 amino acid lengths of Ro60 that contained the TCR epitope and examined whether the identified epitope was properly selected and presented by HLAs (Fig. 3A). After the coculture with 293T cells transfected with the corresponding HLA alleles and the CD74-Ro60 fusion vector, all reporters reacted when the fusion vector contained an epitope sequence (Fig. 3B). The percentage of green fluorescent protein (GFP)–positive reporters was similar when the sequence contained in the fusion vector was in the range of 40 to 120 amino acid lengths and was comparable with that observed with the addition of peptides instead of fusion vectors. These results indicated that identified epitopes could be processed and presented to the HLAs.

Another validation was performed with full-length Ro60 protein and antigen-presenting cells (APCs) under more physiological conditions. One of the differences between healthy individuals and serum anti-Ro60 antibody-positive SjD is that patients have abundant anti-Ro60 antibodies in their serum, so when Ro60 is released from dead cells, autoantibodies immediately bind to Ro60 and form immune complexes. It is known that such immune complexes are efficiently taken up by APCs and some of them are presented as antigens (*27*). Therefore, we prepared immune complexes containing Ro60 and then phagocytosed them by APCs to examine whether the epitopes we identified in this study were presented.

**Fig. 4.**
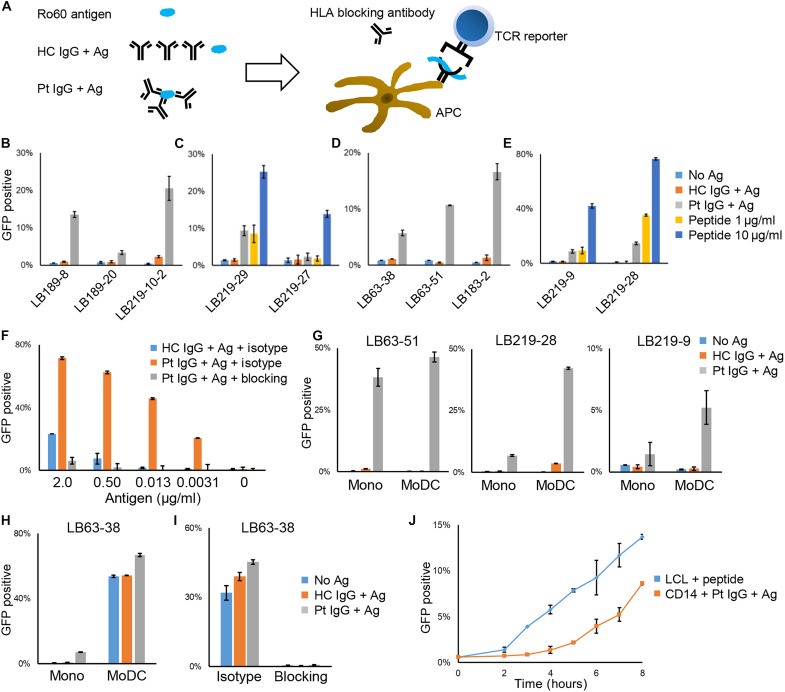
Validation of identified epitopes using full-length autoantigen. (**A**) Schema of antigen (Ag) presentation experiments. APCs and reporter cells were cocultured with full-length Ro60 protein, serum IgG from healthy control (HC IgG) or from patient (Pt IgG), and HLA blocking antibody. (**B** to **E**) The proportion of GFP-positive reporter cells after overnight coculture with HLA-matched PBMCs and a mixture of Ro60 and HC or Pt IgG. For selected TCRs, we compared responses with those induced by the corresponding peptide (C and E). (**F**) The proportion of GFP-positive LB183-2 reporters after overnight coculture with HLA-matched PBMCs from HC and the indicated concentration of Ro60 and its 10× concentration of IgG. Blocking or isotype control antibody was used at 10 μg/ml. (**G** and **H**) The proportion of GFP-positive reporter cells after overnight coculture with HLA-matched peripheral monocytes (Mono) or MoDCs with a mixture of Ro60 and HC or Pt IgG. (**I**) The proportion of GFP-positive LB63-38 reporter cells after overnight coculture with HLA-matched MoDC and a mixture of Ro60 and HC or Pt IgG in the presence of 10 μg/ml of HLA blocking antibody or isotype. (**J**) Time course of GFP positivity of LB63-51 reporter cells cocultured with LCL that had been preincubated and cocultured with 50 μg/ml of antigen peptide for at least 1 hour, or with peripheral monocytes and a mixture of Ro60 and Pt IgG are shown. Data are shown as the median ± SD of duplicates. Unless otherwise indicated, the Ro60 protein and purified IgG were used at 2 and 20 μg/ml, respectively.

Immune complexes were prepared by mixing purified immunoglobulin G (IgG) from patient sera with full-length Ro60 protein. These complexes were then evaluated using a CD16 reporter cell line, which induces GFP expression in proportion to immune complex engagement, enabling relative quantification of immune complex levels (*28*). First, we confirmed that when IgG from each patient serum was mixed with Ro60, the amount of immune complexes formed reflected the anti-Ro60 antibody titer of that serum (fig. S5A). Then, we cocultured the reporter cells with APCs and immune complexes using IgG from various patients (Fig. 4A). Initially, we used peripheral blood mononuclear cells (PBMCs) as APCs. As shown in Fig. 4 (B to E), after overnight coculture, all reporters reacted to the addition of a mixture of patient IgG and Ro60 protein (immune complex), with varying response magnitudes. For selected TCRs, immune complex–induced responses were comparable to those elicited by corresponding peptide stimulation at 1 μg/ml (Fig. 4, C and E). The reactivity of the TCR reporter correlated with that of the CD16 reporter and the anti-Ro60 antibody titer of the IgG used, irrespective of the TCR type (fig. S5, B to D), indicating a dose-dependent relationship between immune complex quantity and peptide presentation level. To investigate the difference in antigen presentation efficiency between native antigen and immune complexes, we used the LB183-2 reporter. We found that immune complexes induced a markedly stronger antigen presentation compared with native antigen alone, with an antigen concentration 64-fold lower than that required for native antigen stimulation (Fig. 4F). These findings suggested that serum autoantibodies can substantially enhance the efficiency of self-antigen presentation in vivo.

Next, we used peripheral monocytes or monocyte-derived dendritic cells (MoDCs) as APCs. In the same coculture, the reporter reacted more efficiently to immune complexes on monocytes than on PBMCs and on dendritic cells (DCs) than on monocytes (Fig. 4, G and H). Unexpectedly, the LB63-38 reporter showed a high response in coculture with MoDCs, without the addition of immune complexes or even antigens. This response was blocked by anti-HLA-DR blocking antibody (Fig. 4I), suggesting that the epitope of LB63-38 was derived from endogenous Ro60 and constitutively displayed on DCs.

We next conducted a time-course experiment to determine the kinetics of antigen presentation. Reporters were cocultured either with peptide-pulsed LCLs prepared in advance or with monocytes immediately following the addition of immune complexes. We found that it took ~3 hours for the immune complexes to be processed and for the peptides to be presented on the surface of APCs (Fig. 4J). These results indicated that the T cell epitope identified in this study could be efficiently presented by APCs in the presence of anti-Ro60 antibodies.

### Validation of autoreactive TCR function using primary CD4^+^ T cells

**Fig. 5.**
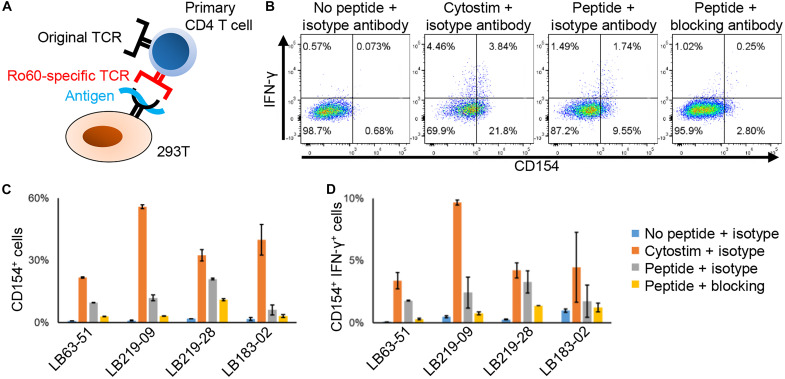
Functional validation of identified TCRs. (**A**) Schema of TCR stimulating experiments using primary CD4^+^ T cells. Ro60-specific TCR-overexpressing primary CD4^+^ T cells were cocultured with class II HLA–expressing 293T with or without the corresponding peptide. After 6-hour coculture, cells were analyzed by intracellular staining. (**B**) Representative results of intracellular staining of CD154 and IFN-γ, and (**C**) the proportion of CD154^+^ cells or (**D**) CD154^+^IFN-γ^+^ cells among LB63-51, LB219-09, LB219-28, and LB183-02 overexpressed primary CD4^+^ T cells are shown. Blocking or isotype control antibody was used at 10 μg/ml. Data are shown as the median ± SD of duplicates.

As it is known that TCRs require an optimal affinity for the HLA/peptide complex (neither too high nor too low) to trigger optimal antigen recognition, we next examined whether the TCR identified in this study would also provide the appropriate stimulation on primary T cells. Primary CD4^+^ T cells from healthy individuals were forced to express Ro60-specific TCR by retroviral transfection and cocultured with the corresponding class II HLA–expressing 293T cells and epitope peptides (Fig. 5A). After a 6-hour coculture, CD4^+^ T cells rapidly expressed CD154 in the presence of the corresponding peptide or the superantigen (positive control), and a part of CD154^+^ cells also coexpressed interferon-γ (IFN-γ), whereas they did not in the absence of the peptide or in the presence of the anti-HLA blocking antibodies (Fig. 5, C and D). These results indicated that the TCRs identified in this study were functional and capable of activating primary T cells in vivo.

### Characteristics of Ro60-specific T cells

**Fig. 6.**
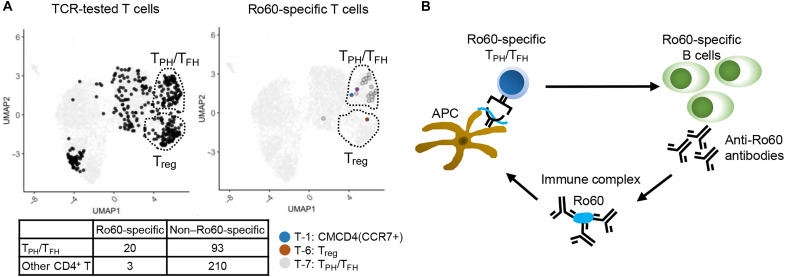
Characteristics of Ro60-specific T cells in salivary gland of SjD. (**A**) TCR-tested T cells and Ro60-specific T cells are plotted on UMAP shown in Fig. 1C. The colors indicate the classification of T cell clusters. The table shows the number of cells analyzed for antigen specificity, categorized by T_PH_/T_FH_ identity and Ro60 specificity. (**B**) The pathological loops in the salivary glands suggested by the results of this study are shown. Anti-Ro60 antibody-producing B cells produce autoantibodies that bind to Ro60 derived from dead cells and form immune complexes. The immune complexes are efficiently taken up by APCs, and Ro60-derived peptides are presented by various class II HLAs. Ro60-specific T_PH_/T_FH_ cells are activated by these HLA/peptide complexes and help anti-Ro60-producing B cells to produce antibodies.

Next, we combined the results of antigen specificity of the TCRs with the results of CD4^+^ T cell subclusters from scRNA-seq analysis to examine the characteristics of the Ro60-specific T cells in the salivary glands. As shown in Fig. 6A, Ro60-specific T cells were highly enriched in the T_PH_/T_FH_ cluster [*P* < 0.0001, Fisher’s exact test comparing Ro60 reactivity and cluster identity (T_PH_/T_FH_ versus others)], accounting for 7.1% of the analyzed CD4^+^ T cells and representing 17.7% of the T_PH_/T_FH_ population. Our results indicated that the Ro60-specific B cells and Ro60-specific T_PH_/T_FH_ cells orchestrated the pathological loop in the salivary gland of patients with SjD, as shown in Fig. 6B.

### Identification of Ro60-specific TCRs from Caucasian populations

HLA haplotypes greatly differ depending on race and geographic region. To confirm whether the Ro60-specific T cells were found in the salivary glands of SjD in populations with different HLA haplotypes, we generated another 47 TCR reporters based on previously reported TCRs derived from salivary glands of Caucasian patients with SjD (*29*) and examined its antigen specificity.

**Fig. 7.**
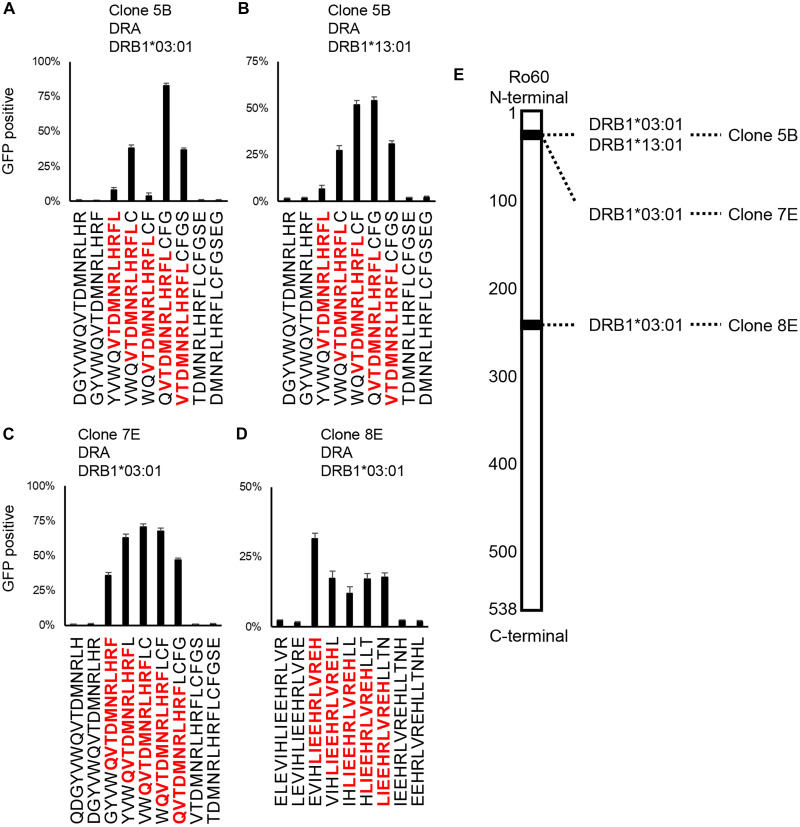
Identification of T cell epitopes in Caucasians. Determination of the core epitope recognized by TCRs of salivary gland CD4^+^ T cells reported in the previous study (*29*). The TCR reporter cells of (**A** and **B**) clone 5B, (**C**) clone 7E, and (**D**) clone 8E were cocultured with 293T cells expressing the indicated HLA-DR and with serial overlapping 15-mer peptides. The core epitopes are shown in red. Data are shown as the median ± SD of duplicates. (**E**) The distribution of T cell epitopes in the Ro60 molecule and the corresponding HLA and TCR are shown. The black areas indicate T cell epitopes.

Among the 28 TCRs derived from six serum anti-Ro60 antibody-positive patients and 16 TCRs from four antibody-negative patients, three Ro60-specific TCRs were identified exclusively from the antibody-positive group (Fig. 7, A to D). The position of epitopes and corresponding HLA alleles are summarized in Fig. 7E. These results suggested that the coordination of Ro60-specific B cells and T cells in the salivary glands is the underlying pathogenesis of anti-Ro60 antibody-positive SjD, independent of race and HLA haplotype. The sequences of all Ro60-specific TCRs, corresponding HLAs, and corresponding antigens are summarized in Table 2.

**Table 2.** Summary of TCR sequences, HLA alleles, and core epitope positions.

TCR name	TRAV	TRAJ	TRA junction	TRBV	TRBJ	TRBD	TRB junction	HLA allele	Core
LB189-8	8-3*01	34*01	CAVFLYNTDKLIF	6-5*01	2-5*01	2*01	CASSYRRKETQYF	DRA1*01:01 + DRB1*14:06	27–35
LB189-20	13-1*02	37*02	CAAQSPSSNTGKLIF	18*01	1-1*01	1*01	CASSPTRDRVGAFF	DRA1*01:01 + DRB1*14:06	26–35
LB219-29	4*01	9*01	CLVGDIGGTGGFKTIF	5-1*01	2-7*01	1*01	CASSFEQGGNYEQYF	DRA1*01:01 + DRB1*14:06	31–36
LB219-10-2	26-1*01	44*01	CIVRVFTGTASKLTF	6-6*01	2-7*01	2*01	CASSSLGYEQYF	DRA1*01:01 + DRB1*14:06	28–35
LB219-27	12-2*02	5*01	CAVNNGGRRALTF	2*01	1-2*01	1*01	CASSDQDANYGYTF	DRA1*01:01 + DRB1*14:06	26–37
LB219-9	17*01	30*01	CATDAVDDKIIF	3-1*01	2-2*01	1*01	CASSQFGQGELFF	DRA1*01:01 + DRB1*15:01	32–41
LB219-28	35*02	40*01	CAGLLTSGTYKYIF	5-1*01	2-5*01	1*01	CASSSKQGLGKTQYF	DQA1*01:02 + DQB1*06:02	504–512
LB183-2	9-2*01	5*01	CALSGTDDTGRRALTF	7-2*01	1-5*01	1*01	CASSLKGWDYNQPQHF	DPA1*02:02 + DPB1*05:01	228–237
LB63-51	3*01	5*01	CAVRNTGRRALTF	12-4*01	2-7*01	2*01	CASSYTGAGIHEQYF	DRA1*01:01 + DRB1*15:01	72–82
LB63-38	25*01	37*01	CAGLKLGSGNTGKLIF	2*01	2-1*01	2*02	CASSQSGRPGFF	DRA1*01:01 + DRB1*15:01	525–537
Clone 5B	26-1*01	5*01	CIVRVVTGRRALTF	20-1*03	2-1*01		CSATTSTGGNEQFF	DRA1*01:01 + DRB1*03:01/13:01	27–37
Clone 8E	20*01	13*01	CAVQAIPGGYQKVTF	24-1*01	2-2*01	2*01	CATGEQNTGELFF	DRA1*01:01 + DRB1*03:01	247–257
Clone 7E	8-4*01	17*01	CAVSESGAAGNKLTF	7-9*01	2-7*01		CASSPRDSYEQYF	DRA1*01:01 + DRB1*03:01	26–36

The core sequences of two of three TCRs, clones 5B and 7E, were located in Ro60_26–37_, which were identical to the epitope of five TCRs found in DR*14:06. Furthermore, clone 5B recognizes both DR*03:01 and DR*13:01, the two different DRs possessed by this patient, with the same epitope.

**Fig. 8.**
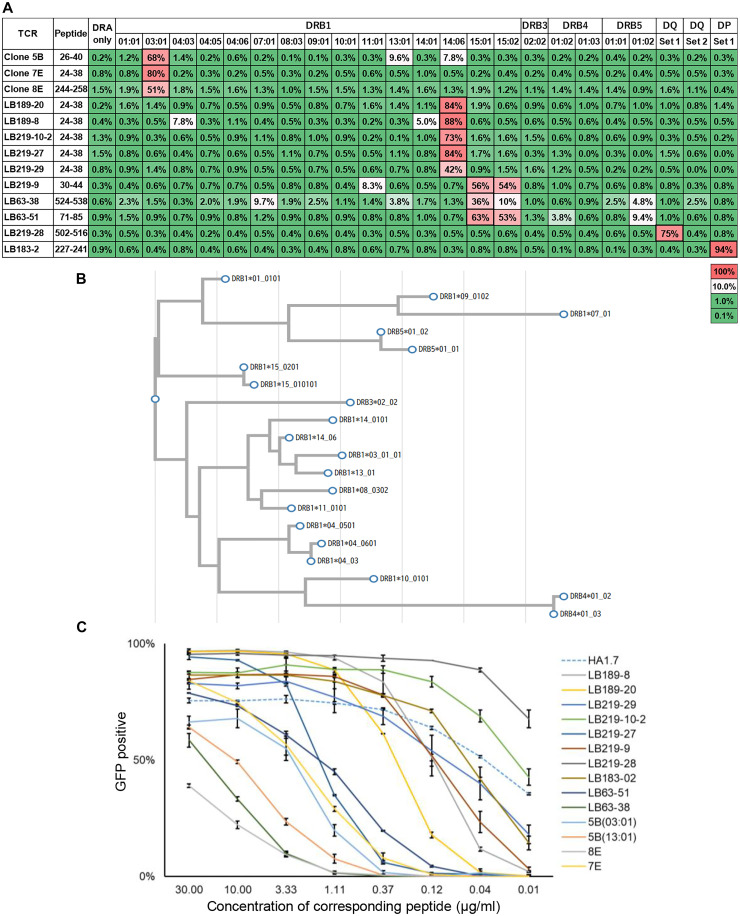
Cross-reactivity and dose-response characteristics of TCR reporters. (**A**) TCR reporters were cocultured with single HLA-DR allele-expressing 293T cells and the corresponding peptide. The reactivity of the TCR reporters is shown. A bold frame indicates the original HLA allele. DQ set 1, DQA1*01:02 + DQA1*01:03 + DQB1*06:02 + DQB1*06:01; DQ set 2, DQA1*03:01 + DQA1*03:02 + DQB1*03:02 + DQB1*03:03; DP set 1, DPA1*01:03 + DPA1*02:01 + DPA1*02:02 + DPB1*02:01 + DPB1*05:01 + DPB1*09:01. (**B**) Homology of amino acid sequences in the extracellular region of the HLA allele is shown in a phylogenetic tree. (**C**) The proportion of GFP-positive reporter cells after overnight coculture with 293T cells expressing the corresponding HLA molecule and pulsed with graded concentrations of corresponding peptide are shown. Data are shown as the median ± SD of duplicates. HA1.7 was used as external control.

To further investigate whether there were other TCRs that could recognize multiple HLA alleles, we cocultured TCR reporters and 293T cells expressing various HLAs in the presence of the corresponding peptides. As shown in Fig. 8A, approximately half of the TCRs were able to react with multiple HLAs with varying strength of reactivity. In particular, DRB1*15:01 and DRB1*15:02, which are close to each other in amino acid sequence phylogenetic analysis (Fig. 8B), cross-reacted well. Cross-reactivity was also observed between DRB1*14:06 and DRB1*14:01, DRB1*15:01, and DRB5*01:02.

Having identified all 13 Ro60-specific TCRs, we next evaluated their functional sensitivity by benchmarking them against the well-characterized TCR HA1.7 [the dissociation constant (*K*_D_) = 37 μM; (*30*)]. Each TCR reporter was cocultured with 293T cells expressing the corresponding HLA molecule and pulsed with graded concentrations of peptide. As shown in Fig. 8C, the Ro60-specific TCRs exhibited a broad range of response strengths—some surpassing HA1.7 and others responding more weakly—demonstrating that self-antigen recognition occurs with diverse functional sensitivities across these clones.

### Comparison of TCR clones between salivary glands and peripheral blood

Next, although limited by a small sample size, we attempted to compare the TCR repertoires between salivary glands and peripheral blood. For two individuals (LB189 and LB219), paired PBMC samples collected closest to the time of salivary gland biopsy were subjected to bulk TCR repertoire analysis and compared with salivary gland–derived TCRs. As shown in fig. S6 (A and B), a small but detectable overlap of TCR clones was observed between salivary gland and peripheral blood samples. Among TCR clones identified in peripheral blood, 0.25 and 0.40% overlapped with salivary gland–derived clones at the clonal level in LB189 and LB219, respectively, corresponding to 0.55 and 1.03% at the estimated cellular level. No clear correlation was observed between the relative frequencies of the shared clones in the salivary gland and peripheral blood compartments (fig. S6C). Most shared clones were more highly enriched in the salivary gland than in peripheral blood, with the exception of two clones in LB219 (fig. S6D), suggesting that a substantial proportion of disease-relevant T cells undergo local clonal expansion within the salivary glands. Notably, one Ro60-specific TCR was detected among the shared clones and exhibited an approximately two-log clonal expansion in the salivary gland compared with peripheral blood.

### Sequence similarity analysis using TCR database

Last, we assessed the similarity between the sequence of Ro60-specific TCRs and that of previously reported antigen-specific TCRs (*31*). Ro60-specific TCRs displayed diverse VDJ gene usage and heterogeneous CDR3 sequences (Table 2), with minimal overlap among clones. A VDJdb search allowing for a single amino acid mismatch within the CDR3 region yielded 15 TCR sequences with partial similarity; however, the majority were restricted by class I HLA alleles, and no matches demonstrated homology across both the TCRα and β chains (fig. S7). No TCRs have been registered with Ro60 as an antigen; therefore, this study identifies human TCRs reactive to Ro60.

## DISCUSSION

In this study, we identified that salivary gland–infiltrating CD4^+^ T cells in patients with SjD, particularly T_PH_/T_FH_ cells, were enriched for reactivity to Ro60. We confirmed that multiple TCRs from different patients responded to the same antigen and that, when the full-length protein was used as the antigen in the form of immune complexes, the relevant peptide was presented by HLA molecules in a manner capable of inducing TCR activation, with some variability in responsiveness among TCRs. Notably, for the TCRs tested, antigen presentation by the full-length protein appeared to induce responses comparable to those observed with peptide pulsing at ~1 μg/ml. Moreover, the antigen-specific TCRs were capable of transmitting activation signals in primary CD4^+^ T cells derived from healthy donors, indicating their functional competence. Collectively, these findings strongly support the notion that Ro60 represents a major target of CD4^+^ T cells within the affected tissue. Previous studies by our group and others have reported an enrichment of Ro60-specific antibody-secreting cells in the salivary glands (*6*–*8*). We have also identified the presence of Ro60-specific B cells in bronchoalveolar lavage fluid from SjD complicated by interstitial lung disease (*9*). When considered together with the current findings, this suggests that Ro60-specific B cells and CD4^+^ T cells form a pathogenic feed-forward loop within the salivary glands and probably other affected organs.

We identified HLA-DRB1*15:01, DQA1*01:02/DQB1*06:02, and DPB1*05:01 as HLA molecules corresponding to Ro60-specific CD4^+^ T cells, all of which showed significantly elevated ORs in serum anti-Ro60 antibody-positive patients with SjD. Although statistical significance was not reached due to its rarity, HLA-DRB1*14:06, which exhibited the highest OR in our study, was also associated with multiple Ro60-specific TCRs. Moreover, Ro60-specific TCRs from previously reported Caucasian cohorts (*29*) were found to be restricted by HLA-DRB1*03:01, an allele known to confer increased disease risk in Caucasian populations (*21*, *22*). These findings suggested that the Ro60-specific TCRs identified in this study may play a pathogenic role in driving disease onset in SjD.

While the identification of T cell epitopes in human autoimmune diseases has advanced for organ-specific autoantigens, such as those in type 1 diabetes and multiple sclerosis, analysis of organ-nonspecific autoantigens in systemic autoimmune diseases, as seen in SjD, has lagged behind. Recently reported examples of organ-nonspecific autoantigens include citrullinated peptides in RA (*32*–*34*) and “neoself” antigens in SLE (*35*), the latter arising due to reduced expression of the invariant chain, leading to aberrant antigen presentation. Although these antigens are derived from self-proteins, they undergo posttranslational modifications or structural alterations that may lead to their misrecognition as non-self. In contrast, Ro60, identified in this study, is a bona fide self-protein that does not undergo posttranslational modifications or structural changes and is expected to be presented as self during the thymic selection of T cells. Nevertheless, Ro60-specific CD4^+^ T cells were significantly enriched in the T_PH_/T_FH_ cluster, rather than in the FOXP3^+^ T_reg_ cluster. This finding indicates the breakdown of immune tolerance and highlights the need for further investigation into the mechanisms underlying this failure.

In recent years, there have been growing efforts to develop antigen-specific immunotherapies (*36*, *37*). These include liposomal formulations containing epitope peptides of pathogenic CD4^+^ T cell and vitamin D to generate of self-antigen-presenting tolerogenic DCs (*38*), peptide-HLA complexes displayed on nanoparticles to induce Tr1 cells by stimulating T cells in the absence of costimulatory signals (*39*), and adoptive transfer therapies using T_reg_ cells that have been cotransfected with both antigen-specific TCRs and FOXP3 to enhance their suppressive function (*40*, *41*). These approaches rely critically on knowledge of disease-specific TCR sequences and their corresponding HLA-peptide complexes. Validated Ro60 epitopes and functional TCRs shown in this study will greatly advance the development of antigen-specific immunosuppressive therapy in SjD. Notably, we identified a TCR that responded to both HLA-DRB1*03:01 and HLA-DRB1*13:01 alleles in the same patient, as well as TCRs reactive to HLA-DRB1*15:01 that also recognized HLA-DRB1*15:02, which differs by only one amino acid in the extracellular domain. Although TCRs are classically thought to require recognition of one specific HLA molecule and one specific peptide, our findings suggest that certain TCRs may recognize shared structural motifs across multiple HLA alleles, as also demonstrated in the context of cancer immunity (*42*). This raises the possibility that a single TCR-based therapy could be applicable to patients carrying structurally similar HLA alleles.

A previous insightful study using HLA-DRB1*15:02 transgenic mice immunized with human Ro60 identified T cell epitopes within amino acids 101 to 185 and 381 to 475 (*43*). In contrast, our DRB1*15:02-reactive TCRs recognized Ro60 regions amino acids 30 to 44, 71 to 85, and 524 to 538 (Fig. 8A), which do not coincide with the previously reported regions. This difference may reflect the limited number of human TCRs analyzed in our study, but it may also arise from differences between the human and mouse TCR repertoires. These findings further underscore the need for continued accumulation of human patient–derived data to elucidate the molecular basis of autoreactive T cell responses.

The frequency of Ro60-specific CD4^+^ T cells within the T_PH_/T_FH_ cluster was ~23%, comparable with ~30% of infiltrating B cells in salivary glands. Given that T_PH_/T_FH_ cells play a key role in providing help to autoreactive B cells, targeting Ro60-specific CD4^+^ T cells may also suppress Ro60-specific B cells. Combined with the clinical recommendation of rituximab, a B cell–depleting therapy for severe cases of SjD (*44*), therapeutic strategies targeting Ro60-specific T cells may provide promising efficacy without suppressing the immune system other than autoimmunity.

This study has several limitations. We performed HLA frequency analysis to identify alleles enriched in Japanese patients with SjD; however, the sample size was limited for a robust disease-association study. Therefore, HLA alleles with low frequency or modest ORs could not have been detected. Among the identified alleles, we successfully isolated TCRs restricted by DRB1*15:01, DQA1*01:02/DQB1*06:02, and DPB1*05:01, which had relatively high ORs, but no TCRs were found corresponding to DRB1*08:03. One contributing factor may be the limited number of TCRs obtained from some samples, probably due to the use of small-volume biopsy specimens collected to minimize invasiveness. Moreover, although various CD4^+^ T cell subsets were present in the salivary glands, Ro60-specific T cells were almost exclusively found in the T_PH_/T_FH_ cluster. The antigen specificities of CD4^+^ T cells in other subsets remain unclear. Several exogenous factors have been implicated in the pathogenesis of SjD, including oral commensal bacteria (*16*), gut microbiota (*45*), and Epstein-Barr virus (EBV) (*46*, *47*). While it has been suggested that immune responses to exogenous peptides could cross-react with Ro60, a search of publicly available TCR databases did not reveal any known TCR pair sequences specific for exogenous antigens similar to the TCRs identified in this study. As discussed earlier, how immune tolerance is broken to allow a pathogenic response against Ro60 remains an important question for future investigation.

In conclusion, we provide the first direct molecular evidence that CD4^+^ T cells and B cells infiltrating the lesions of systemic autoimmune diseases coordinate immune responses against a shared antigen. This approach is applicable to other systemic autoimmune diseases and offers important insights for the development of future antigen-specific therapies.

## MATERIALS AND METHODS

### Experimental design

This study is an experimental investigation using clinical samples from patients, conducted to test the hypothesis that CD4^+^ T cells in SjD recognize the same autoantigens as B cells. Paired peripheral blood and labial salivary glands from patients with SjD were collected at Keio University Hospital. This study was approved by the Institutional Review Board of Keio University School of Medicine (20221186) and conducted in compliance with the tenets of the Declaration of Helsinki. Written informed consent was obtained from all participating individuals. The classification of SjD was conducted according to the classification criteria for primary SjD established in 2016 (*48*). DNA was extracted from peripheral blood using NucleoSpin Blood QuickPure (Takara Bio, Shiga, Japan), and HLA typing was performed by the HLA laboratory (Kyoto, Japan). Peripheral bloods were also used for generating LCLs. Salivary gland samples were used for immunostaining and single-cell analysis as described later.

### Generating LCL

LCLs were generated as previously described (*49*). In brief, B95-8 cells (JCRB, Osaka, Japan) in cRPMI medium [RPMI 1640 medium (Thermo Fisher Scientific, MA, USA) supplemented with 2 mM l-glutamine, 1% nonessential amino acids, 55 μM 2-mercaptoethanol, 1 mM sodium pyruvate, penicillin (100 U/ml), streptomycin (100 U/ml), and 10% fetal bovine serum] at 1 × 10^6^ cells/ml were stimulated with phorbol 12-myristate 13-acetate (20 ng/ml) for 1 hour. Cells were washed three times and further cultured for 3 days. The EBV containing supernatant was collected by centrifugation at 600*g* for 10 min at 4°C, filtered through a 0.45-μm filter, aliquoted, and stored at −80°C until use.

PBMCs were isolated using density gradient centrifugation (Lymphoprep, Axis-Shield, Oslo, Norway) and suspended in cRPMI medium at 2 × 10^6^/ml with cyclosporin A (200 ng/ml; FUJIFILM Wako, Osaka, Japan) at 37°C for 1 hour. The EBV-containing supernatant was thawed and added to the cells at a 1/10 dilution. After ~2 weeks of culture, a half medium change was performed when clumps of cells became visible, and after the cells became stably increasing, passages were made twice a week.

### Processing of salivary gland samples and TCR analysis

TCR analysis methods for salivary glands from each case are listed in table S1. Single-cell suspensions from labial salivary gland tissues were prepared as previously described (*10*). In brief, a part of the salivary gland tissue was mechanically and enzymatically digested and filtered through a 40-μm cell strainer (Greiner Japan, Tokyo, Japan).

Samples from LB183, LB189, LB215, LB216, LB219, and LB221 were analyzed using the Chromium Next GEM Single Cell 5′ Kit (10x Genomics, CA, USA) according to the manufacturer’s protocol. In brief, single-cell suspension was stained using fluorochrome-conjugated antibodies against CD45, CD3, CD4, CD8, CD19, CD38, and CD326 for 20 min at 4°C, washed, resuspended in sorting buffer containing 7-aminoactinomycin D (7-AAD), and used for cell sorting. T cells (7AAD^−^CD326^−^CD45^+^CD3^+^CD19^−^), B cells (7AAD^−^CD326^−^CD45^+^CD3^−^CD19^+^), other CD45^+^ cells (7AAD^−^CD326^−^CD45^+^CD3^−^CD19^−^), and salivary gland epithelial cells (7AAD^−^CD326^+^CD45^−^) were sorted using a FACS Aria III system (BD Biosciences, NJ, USA), mixed, and used for single-cell analysis.

Samples from LB63 and LB65 were used for sorting based on TCR analysis. Single-cell suspensions were stained as described above and 32 CD4^+^ T cells (7AAD^−^CD326^−^CD45^+^CD3^+^CD19^−^CD4^+^CD8^−^) were sorted using a FACS Aria III from LB63 and LB65, respectively, and the Smart-Seq2 (*50*) protocol was performed, modifying only the polymerase chain reaction (PCR) step to 23 cycles for single-cell cDNA generation. The variable regions of TCRα and TCRβ gene transcripts were amplified by PCR according to previously published methods (*51*) with some modifications. In brief, PCR was performed with a total volume of 20 μl containing 1 μl of the cDNA library, 300 nM of each primer, and 10 μl of KAPA HiFi HS ReadyMix (KAPA Biosystems, MA, USA). The cycling parameters were as follows: 95°C for 3 min; 30 cycles at 98°C for 20 s, 65°C for 15 s, and 72°C for 30 s; and 72°C for 1 min. PCR products were electrophoresed on a 0.8% agarose gel, and targeted bands were extracted from the gel using the Gel/PCR purification kit (Favorgen, Taiwan) and sequenced (Eurofins, Tokyo, Japan). A list of the primers used is given in data S1.

### B cell receptor analysis

B cell receptor analysis methods for salivary glands from each case are listed in table S1. Salivary gland–derived B cell reactivity of the samples from LB183, LB189, and LB216 were analyzed as previously described (*6*, *10*) with minor modifications. In brief, the antibody-secreting cells from salivary glands (7AAD^−^CD3^−^CD4^−^CD8^−^CD326^−^CD19^+^CD38^high^) were sorted into 5 μl of RLT buffer (QIAGEN, Venlo, Netherlands) supplemented with 1% of 2-mercaptethanol (Merck, Darmstadt, Germany). RNA was purified with 11 μl of RNAClean XP (Beckman Coulter, CA, USA) and eluted with modified Smart-Seq2 lysis buffer (2.3 μl of nuclease-free water, 1 μl of oligo-dT primer, and 1 μl of deoxyribonucleotide triphosphates mix). RNA extension, reverse transcription, 23 cycles of PCR, and cDNA purification were performed according to Smart-Seq2. The variable regions of IgH, Igλ, and Igκ gene transcripts were amplified and cloned into expression vectors containing the corresponding constant regions of each immunoglobulin using NEBuilder HiFi DNA Assembly (New England Biolabs, MA, USA) as previously described (*6*). Antibodies were produced using the Expi293 Expression System (A14635, Thermo Fisher Scientific), and their reactivity against Ro60 was examined by antigen-binding bead assay as previously described (*6*).

Salivary gland–derived B cell reactivity of the samples from LB183, LB189, LB215, LB219, and LB221 was analyzed by immunostaining using autoantigen as previously described (*10*). In brief, a part of the tissue samples was encapsulated in Tissue-Tek O.C.T. Compound (Sakura Finetek, Osaka, Japan). Ro60-biotin was premixed with Alexa Fluor 555 (AF555)–conjugated streptavidin (Thermo Fisher Scientific). From OCT-embedded salivary gland tissues, 4-mm sections were prepared using a cryostat, sections were fixed in acetone for 10 min, blocked with 5% bovine serum albumin (BSA) and 10% goat serum in phosphate-buffered saline (PBS) for 10 min at room temperature (RT), and incubated with Ro60-AF555 (5 μg/ml) and anti-CD138 antibody (5 μg/ml) for 60 min at RT. After washing, the slides were incubated with an Alexa Fluor 594–conjugated anti-mouse IgG antibody for 30 min at RT. After washing, the slides were mounted with VECTASHIELD Mounting Medium with 4′,6-diamidino-2-phenylindole (Vector Laboratories, CA, USA) and observed using an LSM 980 microscope (ZEISS, Oberkochen, Germany).

### Generation of retrovirus for TCR expression

TCR expression vectors were generated from the TCRs of LB63 and LB65, in which both α and β chains could be sequenced, and from the TCRs of LB183, LB189, LB215, LB216, LB219, and LB221, in which clonal expansion were observed (detected in at least three cells in LB219 and two cells in other samples). The variable region sequences of the TCRα and TCRβ chains were amplified by PCR or synthesized (Genscript, NJ, USA) and inserted into the pMX-TCRβ-furin-SGSG-P2A-TCRα-furin-SGSG-P2A-NGFR vector (*52*) using NEBuilder or a Gibson Assembly HiFi Cloning Kit (Thermo Fisher Scientific). The vectors were cloned and amplified using NEB stable (C3040I, New England Biolabs), sequenced, and used for producing retroviruses with Plat-GP cells (RV-103, CELL BIOLABS, CA, USA), following the manufacturer’s protocol. In addition, expression vectors were also generated from the TCR sequences of CD4^+^ T cells that had been clonally expanded in the salivary glands of European and American patients with SjD (*29*).

### Generation of TCR reporter cells

A TCR reporter cell line was generated by stepwise gene transfers into the Jurkat-β-del cell line (JCRB0147, JCRB Cell Bank, Osaka, Japan) as shown in fig. S8. First, Jurkat-β-del cells were infected with lentivirus containing 6× NFAT (nuclear factor of activated T cells) response element, a minimal promoter, and GFP as the reporter gene with a puromycin-resistant gene (VectorBuilder Inc., IL, USA) (*53*) and selected using puromycin (4 μg/ml). Then, cells were infected with a pMX retrovirus encoding full-length human CD4, which had been cloned from cDNA prepared from human PBMCs using NEBuilder HiFi DNA Assembly. CD4^high^GFP^−^ cells were sorted using a FACS Aria III system and cloned by limiting dilution. Last, cells were infected with one of the patient-derived TCR expression retroviruses, and TCRα/β^high^CD3^high^NGFR^high^GFP^−^ cells were sorted twice. All viral infections were performed using the following protocol. A 24-well culture plate was coated with RetroNectin (50 μg/ml; Takara Bio) in PBS overnight, washed twice using PBS, and blocked with 2% BSA in PBS for 30 min. After discarding the blocking solution, virus solutions were added into the dish, and centrifuged at 2000*g* at 32°C for 2 hours. After discarding the solution, 4 × 10^5^ of cells were added to the dish, cultured at 37°C for 2 or 3 days, and used for cell sorting.

### Generating HLA- or HLA-peptide complex–expressing 293T cells

Total mRNAs were extracted from LCL using NucleoSpin RNA (Takara Bio), and cDNAs were generated using the ReverTra Ace RT kit (TOYOBO, Osaka, Japan). The sequences of HLA-DRA, DRB, DQA, DQB, DPA, DPB, and β2 microglobulin were amplified by PCR from LCL-derived cDNA or synthesized (Genscript) and inserted into the pcDNA3.4 vector (Thermo Fisher Scientific). CD74-antigen fusion vectors were generated by combining the CD74 sequence (318 base pairs from ATG) and partial Ro60 sequence (corresponding to 1 to 40 amino acids, 1 to 80 amino acids, 1 to 120 amino acids, 61 to 140 amino acids, 301 to 380 amino acids, and 481 to 538 amino acids) with a stop codon.

293T cells were obtained from the RIKEN BioResource Center (RCB2202, Tsukuba, Japan). The expression vectors were transiently transfected into 293T using Polyethylenimine Max (Polyscience, PA, USA). After 2 days of transfection, cells were used as APCs for coculture.

### TCR epitope determination assay

Peptides from Ro60 were synthesized at Genscript at >80% purity, dissolved in dimethyl sulfoxide at 10 mg/ml, and stored at −80°C. For coculture experiments, 5 to 10 × 10^4^ TCR reporter cells and LCL or HLA-transfected 293T cells were cocultured in U-bottomed 96-well dishes with single peptide (10 μg/ml), peptide mixture, or 1/100 volume of CytoStim (Miltenyi Biotec, Gladbach, Germany) as positive control, and with blocking antibody (10 μg/ml) against HLA-DR, HLA-DQ, HLA-DP, and HLA-ABC for blocking experiments. After overnight coculture, the GFP expression of live reporter cells (7AAD^−^CD4^+^NGFR^+^) was analyzed using the FACS Verse system and FlowJo software (BD).

### Confirmation of immune complex formation using CD16 reporter cells

CD16 reporter cells were generated as previously reported with minor modifications (*28*). Puromycin-selected Jurkat-β-del cells containing GFP under NFAT control as described above were infected with pMX retrovirus containing human *CD16* (F176V) and *FCER1G*. CD16^high^GFP^−^ cells were sorted using a FACS Aria III system and cloned by limiting dilution. Full-length Ro60 protein was purchased from BBI Solutions (ME, USA). IgG was purified from patient serum or from healthy control (HC) negative for serum autoantibodies using Ab-Capcher MAG2 (protenova, Kagawa, Japan) according to the manufacturer’s protocol. The reactivity of purified serum IgG against Ro60 was examined by antigen-binding bead assay, as described above. Immune complexes were prepared at 10-fold higher concentrations by mixing serum IgG (200 μg/ml) with Ro60 protein (20 μg/ml) for at least 1 hour, and one-tenth of this mixture was then added to the CD16 reporter cultures. After overnight coculture, the proportion of GFP-positive reporter cells, which reflects the relative amount of immune complexes, was measured using a FACS Verse system.

### Validation of TCR epitopes using Ro60-containing immune complex

To isolate CD14^+^ cells, frozen PBMCs were thawed rapidly in a water bath and mixed with 1 ml of prewarmed RPMI 1640 medium containing 1 μl of Benzonase (Merck). After centrifugation at 500*g* for 5 min, supernatants were removed, and cells were suspended in prewarmed cRPMI medium for 30 min at 37°C. PBMCs were counted, and CD14^+^ cells were enriched using human CD14 microbeads (Miltenyi Biotec) according to the manufacturer’s protocol. To generate MoDCs, 5 × 10^5^ of CD14^+^ cells were cultured in 0.5 ml of cRPMI medium with 20 ng/ml each of interleukin-4 (IL-4) and granulocyte-macrophage colony-stimulating factor (GM-CSF; BioLegend, CA, USA) in a 24-well plate. On day 3, 0.2 ml of culture medium was removed and 0.5 ml of cRPMI medium with 40 ng/ml each of IL-4 and GM-CSF was added. On day 6, immature DCs were harvested, washed, and maturated overnight with lipopolysaccharide (100 ng/ml) with or without the Ro60-containing immune complex. The HLA haplotypes of HLA-matched PBMCs, monocytes, and monocyte-derived DCs are shown in table S2.

A total of 5 × 10^4^ TCR reporter cells were cocultured in 50 μl of cRPMI medium in 96-well round-bottom plates with either 3 × 10^5^ PBMCs, 5 × 10^4^ CD14^+^ cells, or 5 × 10^4^ MoDCs. Preformed immune complexes, together with either a blocking antibody or an isotype control antibody (final concentration of 10 μg/ml), were added to the cultures. After overnight coculture at 37°C, the proportion of GFP-positive TCR reporter cells was measured using a FACS Verse system.

### Validation of TCR function using primary CD4^+^ T cells

Frozen PBMCs were thawed as described above and counted, and CD4^+^ T cells were enriched using a human CD4^+^ T cell isolation kit (Miltenyi Biotec). K562/OKT4/CD80 cells, which constantly expressed single chain anti-human CD3 antibody and human CD80, as previously described (*52*), were fixed in 0.25% paraformaldehyde (Nacalai Tesque, Kyoto, Japan) for 5 min and washed three times. Next, 8 × 10^6^ CD4^+^ T cells and 1.2 × 10^6^ fixed K562 cells were cocultured in cRPMI medium supplemented with human IL-2 (5 ng/ml; R&D Systems, MN, USA) and IL-15 (10 ng/ml; BioLegend) for 2 days. Cells were harvested and infected with one of the patient-derived TCR expression retroviruses using RetroNectin, as described above. After 2 days of culture, medium was changed to fresh cRPMI medium supplemented with IL-2 (5 ng/ml). After 3 days of culture, cells were harvested, stained with 7AAD and antibodies against CD4 and NGFR. 7AAD^−^CD4^+^NGFR^+^ cells were sorted as TCR-transfected CD4^+^ T cells and further cultured in cRPMI medium with IL-2 (5 ng/ml). The following day, new culture plates were coated with 2 μg/ml of anti-human CD3 antibody in PBS for 1 hour and washed with PBS three times. Sorted CD4^+^ T cells were added to the CD3-coated plate with 1 μg/ml of anti-human CD28 antibody in cRPMI medium with 5 ng/ml of IL-2. TCR-transfected CD4^+^ T cells were cultured with fresh cRPMI medium supplemented with IL-2 (5 ng/ml) every 2 or 3 days for 7 to 10 days until cells had expanded sufficiently for the next experiments. To verify that functional differences were not attributable to altered receptor abundance, we confirmed that the level of cell-surface TCR expression on TCR-transfected primary CD4^+^ T cells was comparable to that of the TCR-reporter cells and the primary CD4^+^ T cells prior to transfection (fig. S9).

The function of the transfected TCRs was confirmed with the following experiments. Ro60-specific TCR-transfected CD4^+^ T cells were mixed with class II HLA-expressing 293T cells under four conditions: isotype control only, isotype control plus CytoStim (positive control), isotype control plus 10 μg/ml of peptide, and blocking antibody plus peptide. Cells were cultured for 3 hours and further cultured with brefeldin A (5 μg/ml) for 3 hours. Cells were harvested, stained with antibodies against CD4 and NGFR, and fixed using Fluorofix (BioLegend) for 20 min. Cells were washed twice with Intracellular Staining Permeabilization Wash Buffer (BioLegend) and stained with antibodies against CD154 and IFN-γ. After the final wash, cells were analyzed using the FACS Verse system.

### Quality control of scRNA-seq data

After removing systematic background noise using CellBender (version 0.3.0) (*54*), gene expression matrices were loaded into R for further quality control. Using the Seurat package (version 5.0.1) (*55*), the preprocessed data matrix was imported using Read10X and transformed to a Seurat object using the CreateSeuratObject function for each sample. Cells that expressed fewer than 200 genes or contained more than 20% of their total unique molecular identifiers mapping to mitochondrial genes were removed. We further removed doublets using scDblFinder (*56*). Log normalization was performed using the NormalizeData function, and the top 2000 most variable genes were identified by a variance stabilizing transformation method in FindVariableFeatures (*57*). Principal components analysis was used for dimensionality reduction using the top 2000 highly variable genes. We corrected batch effects and heterogeneity within samples simultaneously with the HarmonyMatrix function from the Harmony package (*58*). For cell type analysis of T cells, we carried out the same normalization, feature selection, and scaling steps as described here.

### Cell type identification

After preprocessing steps, we constructed shared nearest neighbor graphs derived from the top 20 harmonized principal components and applied graph-based Louvain clustering (*59*) at various resolution levels. To identify broad cell types in salivary glands, we selected the optimized resolution value (=0.1) based on a manual check of the expression of key markers in each cluster to gain the biological interpretations that made the most sense. Specifically, we checked the expression of key markers including *PTPRC*, *CD3E*, *CD19*, *MS4A1*, *CD14*, *NCAM1*, and *EPCAM*. To identify T cells, we included cells coexpressing at least one productive TCR. For T cell–type analysis, we carried out the same graph-based Louvain clustering with the optimized resolution value (=1.0).

### Single-cell TCR receptor profiling

Each sample was analyzed for TCR sequences using the filtered_contig_annotations.csv file generated by CellRanger. We removed cells with multiple heavy chains. The identified TCR sequences, along with their corresponding cell barcodes, were then aligned with cell barcodes from the RNA library of the same sample. For downstream analysis, we included TCR with productive sequences. Clonal calls were performed by combining the nucleotide and gene sequences using “strict” in each function of the scRepertoire package (*60*).

### Phylogenetic analysis of the extracellular domain of HLA-DR allele

Alignment and phylogenetic reconstructions of amino acid sequences of extracellular domains of the HLA-DR allele were performed using the function “build” of ETE3 3.1.3 (*61*) as implemented on GenomeNet (www.genome.jp/tools/ete/). The maximum likelihood tree (ML tree) was inferred using RAxML v8.2.11 running with model PROTGAMMAJTT and default parameters (*62*). Branch supports were computed out of 100 bootstrapped trees.

### Library construction and sequencing for DNA-based bulk TCR repertoire

For two individuals (LB189 and LB219), genomic DNA was extracted using the AllPrep DNA/RNA Kits (QIAGEN) from frozen PBMC samples collected closest to the time of salivary gland biopsy (the same day for LB189 and 2 months later for LB219, with no changes in treatment during the interval). TCR repertoire sequencing libraries were constructed using in-house multiplex PCR assay. Briefly, we amplified rearranged TCRβ chain using TRBV-specific and TRBJ-specific primers in the first round of PCR. The first-round PCR amplification was performed with QIAGEN Multiplex PCR Kit, and the amplicons were purified using 1.8× volume AMPure XP (Beckman Coulter). The sequencing adaptors were added to first-round PCR amplicons in second-round PCR. Second-round PCR was performed with Phusion Plus PCR Master Mixes (Thermo Fisher Scientific). After purification by 1.8× volume AMPure XP and size selection using BluePippin (Sage Science, MA, USA), the libraries were sequenced using 300-cycle (2 × 150 base pairs) MiSeq Reagent Kit v2 (Illumina, CA, USA).

### Bulk TCR repertoire analysis

Clonotypes were identified using MiXCR version 4.6.0 (*63*). To infer tissue-specific clonal expansion, we performed an integrated analysis of TCR clonotypes shared between salivary gland and peripheral blood samples. Shared clonotypes were defined as those with identical V gene, J gene, and CDR3 amino acid sequences. For scRNA-seq data from salivary glands, we focused on CD4^+^ T cells. The clonal proportion in salivary glands was calculated based on cell counts: specifically, the number of cells expressing a specific clonotype divided by the total number of CD4^+^ T cells analyzed. For paired peripheral blood bulk TCR sequencing data, the clonal proportion was derived from read counts, calculated as the read count for each clone divided by the total sum of TCR read counts in the sample. Because the number of detected TCR sequencing reads was substantially lower than the estimated number of T cells in PBMCs, we approximated clonal cell frequencies using read counts, acknowledging that PCR amplification bias may influence absolute quantification. We then compared the relative abundance of these shared clones between the salivary gland and peripheral blood samples for each individual.

### Antibodies

Antibodies used in this study are listed in table S3.

### Statistical analysis

Comparisons of paired samples were analyzed using Wilcoxon signed-rank test. Comparisons of two and multiple categorical variables were analyzed using Fisher’s exact test and Pearson’s chi-square test, respectively. For the HLA allele frequency study, FDR was calculated using the Benjamini-Hochberg procedure with an allele with a positive frequency of more than 10%. Comparisons of continuous variables were performed using the two-sided Wilcoxon test. Correlations between the two variables were analyzed using Spearman’s rank correlation coefficient. Data shown are representative of two or more independent experiments. Duplicates represent technical replicates. All statistical analyses were performed using JMP Pro 17 (JMP Statistical Discovery, NC, USA).
