# Supplementary material for: Identification of a shared antigen linking CD4+ T and B cell pathology in Sjögren’s disease
Source: Sci Adv. 2026 Jun 3;12(23):eaeb2491. doi: 10.1126/sciadv.aeb2491 (PMC13232566; doi:10.1126/sciadv.aeb2491)
Supplement: Supplementary file 1 — Figs. S1 to S9 Tables S1 to S3 Legend for data S1 [file sciadv.aeb2491_sm.pdf]

Science Advances
AAAS

Supplementary Materials for

Identification of a shared antigen linking CD4^{+} T and B cell pathology in Sjögren's disease

Masaru Takeshita *et al.*

Corresponding author: Masaru Takeshita, takeshita.a5@keio.jp

Sci. Adv. **12**, eaeb2491 (2026)
DOI: 10.1126/sciadv.aeb2491

The PDF file includes:

Figs. S1 to S9
Tables S1 to S3
Legend for data S1

Other Supplementary Material for this manuscript includes the following:

Data S1

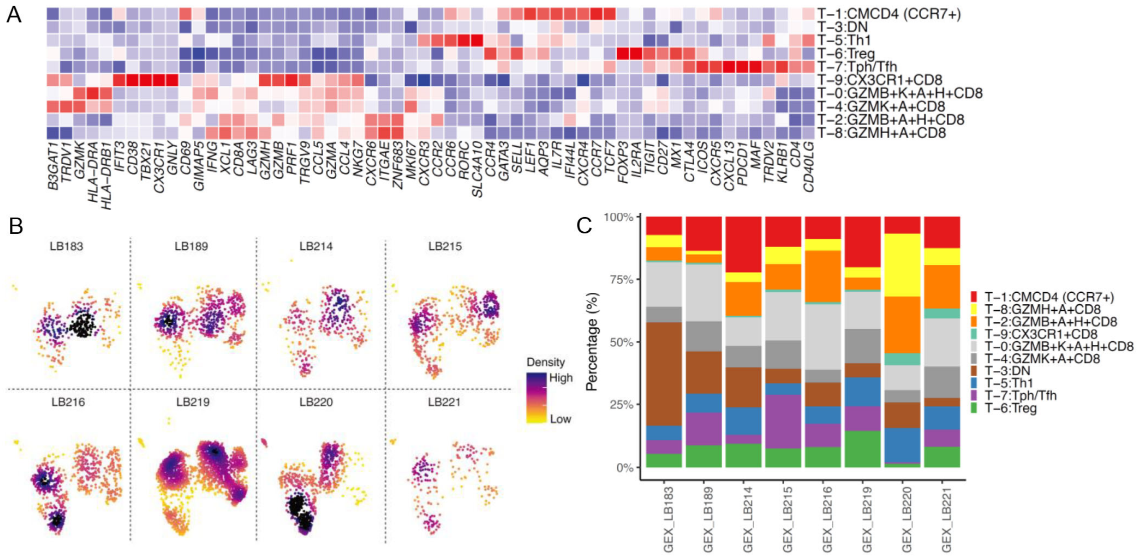

Fig. S1. Identified T cell subclusters in salivary glands of patients with SjD

(**A**) Genes expressed in each T cell subcluster are shown in a heatmap. (**B**) The distribution of T cells and (**C**) the proportion of T cell subclusters from each sample is shown.

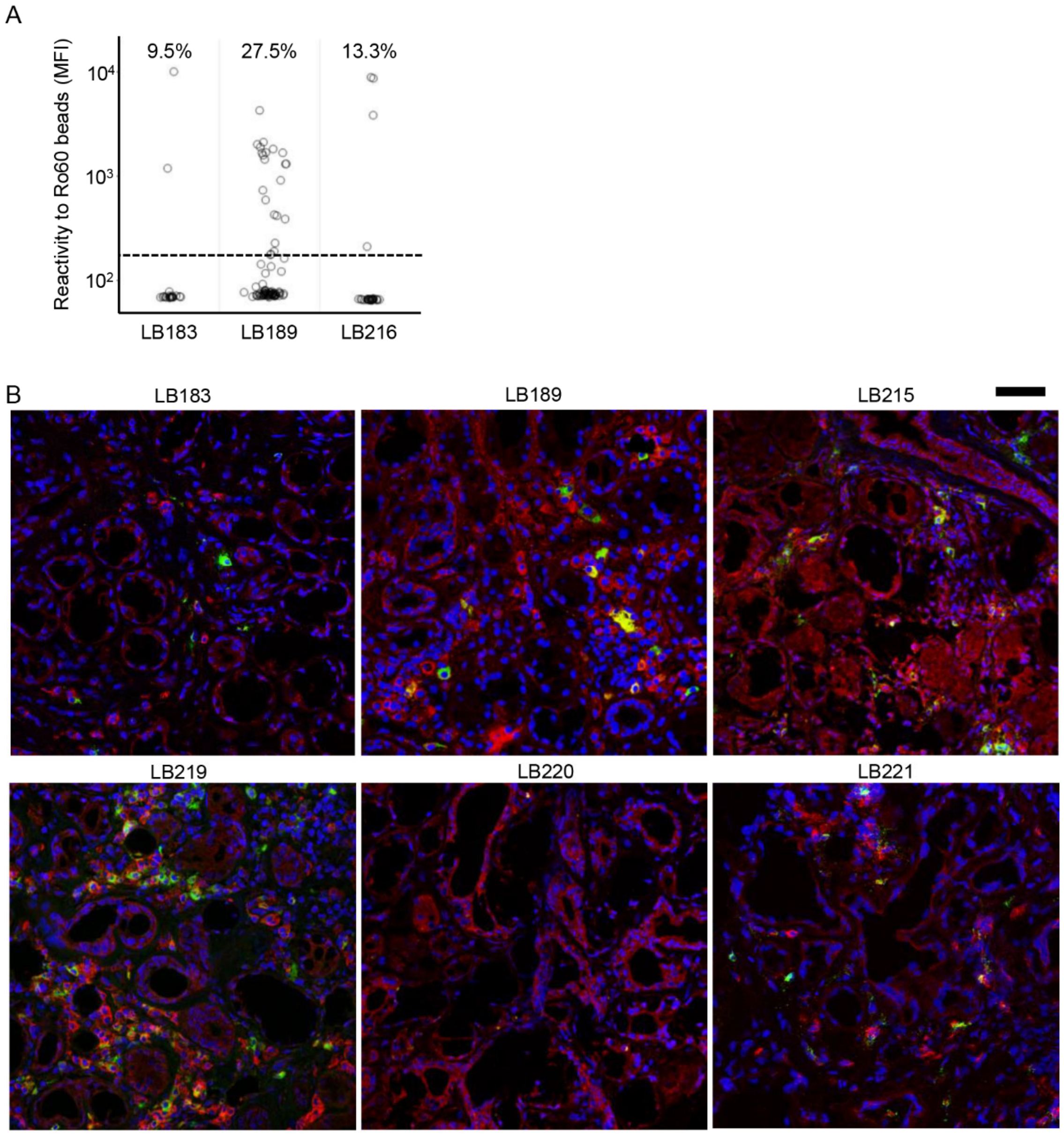

Fig. S2. Detection of anti-Ro60 antibody-producing cells in salivary glands

(**A**) Monoclonal antibodies were generated from salivary glands of LB183, LB189, and LB216 (n = 21, 80, 30). The reactivity of these antibodies against Ro60-binding beads was measured as median fluorescence intensity (MFI) by bead assay. The dashed line indicates the median + 5 × interquartile range cutoff. The proportions of antibodies above the cutoff value are shown. (**B**) Fresh–frozen sections of salivary glands were stained with fluorescently labelled Ro60 (green), anti-CD138 antibody (red, a marker of the antibody-producing cells), and DAPI (blue). Scale bar indicates 50 µm.

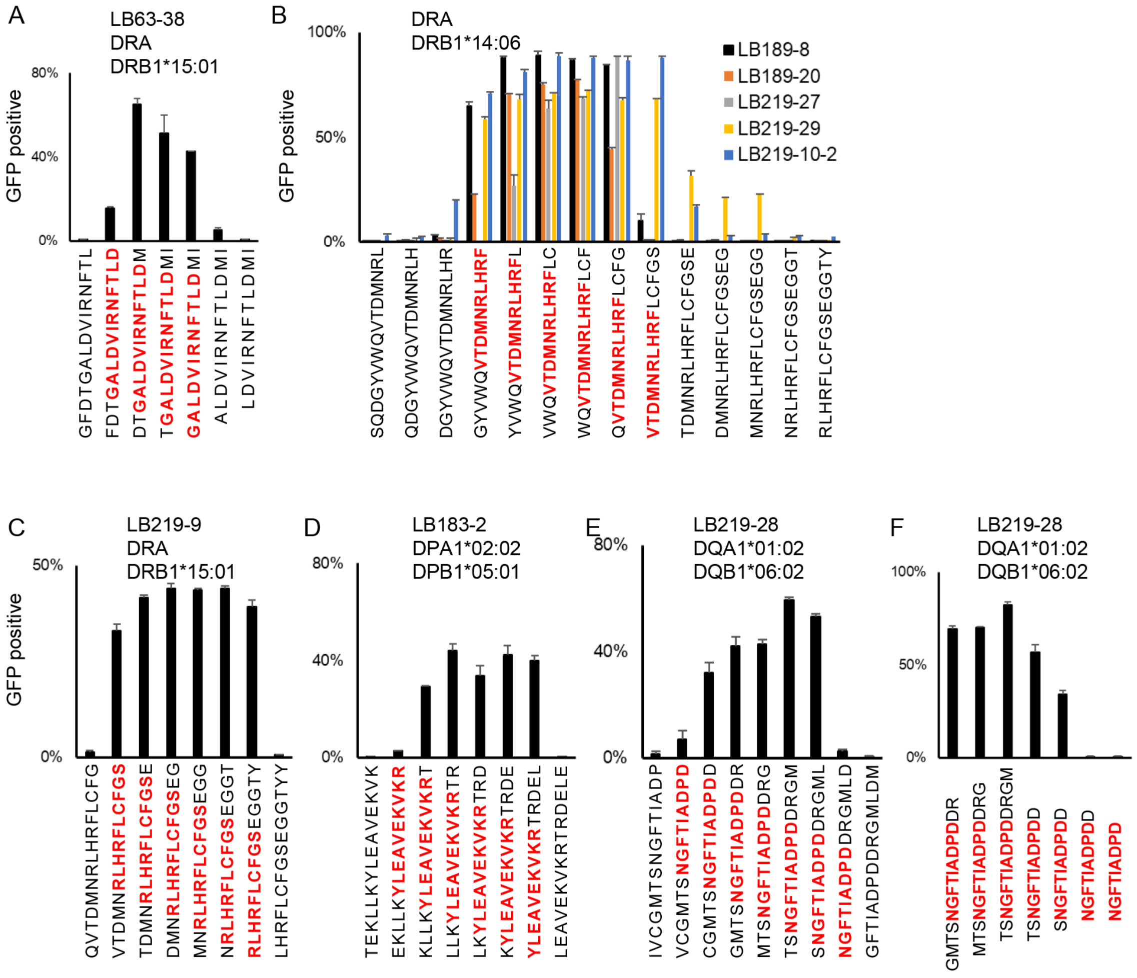

Fig. S3. Identification of T cell epitopes

TCR reporter cells were co-cultured with the corresponding HLA expressing 293T and serial overlapping 15-mer peptides of Ro60. The core epitopes recognized by (**A**) LB63-38, (**B**) LB189-8, LB189-20, LB219-27, LB219-29, and LB219-10-2, (**C**) LB219-9, (**D**) LB183-2, and (**E**) LB219-28 are shown in red. (**F**) The reactivity of LB219-28 to shorter peptides around the core epitope is shown. Data are shown as the median ± SD of duplicates.

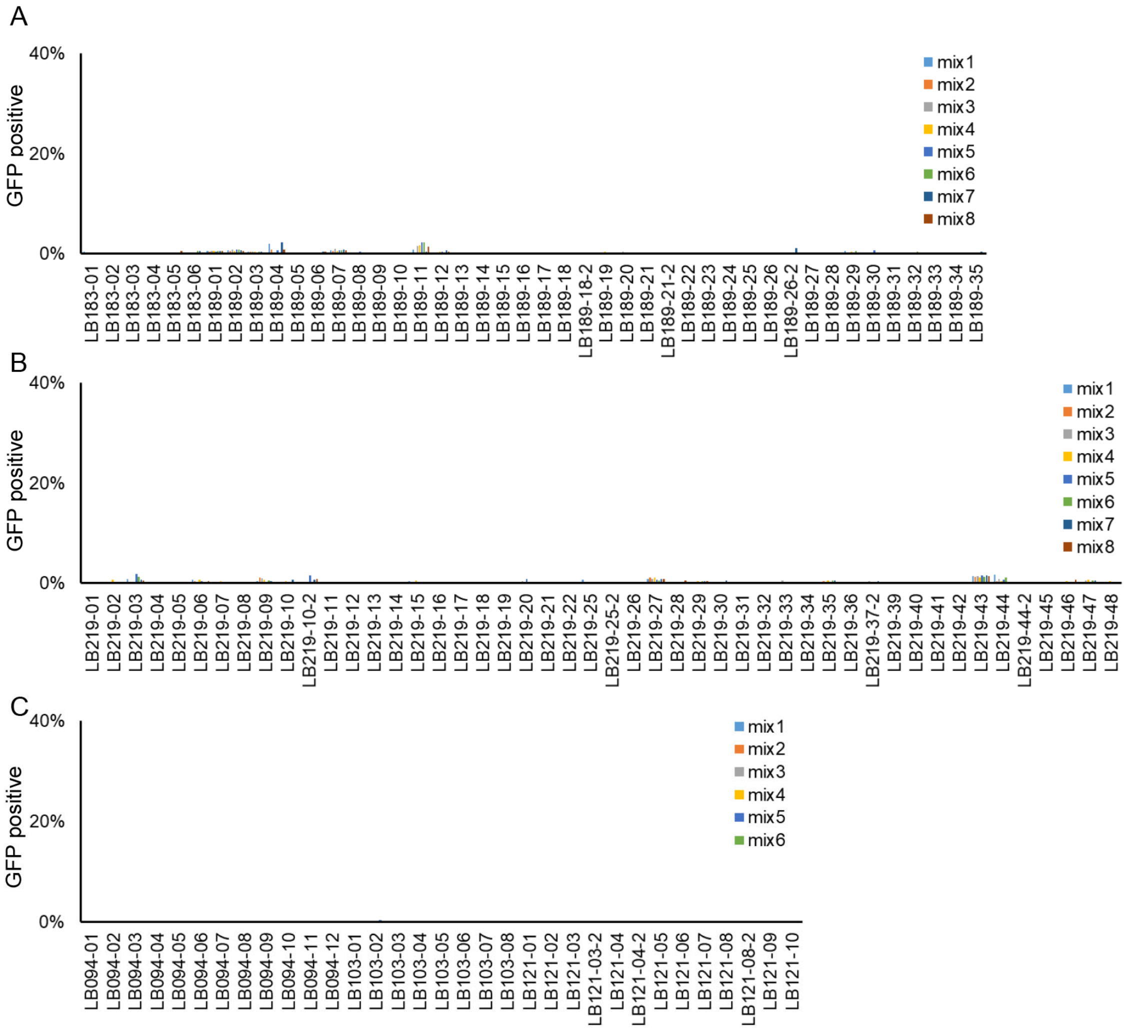

Fig. S4. Confirmation of negative controls

(**A, B**) TCR reporters from LB183, LB189, and LB219 were co-cultured with LCLs from the same patient and a mixture of 10 of 20-mer myeloperoxidase-derived peptides with a 10-amino acid overlap. GFP expression in the reporter cells was measured the following day. (**C**) TCR reporters were generated from previously reporter patients who were seropositive for anti-centromere antibodies but seronegative for anti-Ro60 antibodies (*25*). Reporters were co-cultured with LCLs from the same patient and a mixture of 10 of 20-mer Ro60-derived peptides with a 10-amino acid overlap. GFP expression in the reporter cells was measured the following day.

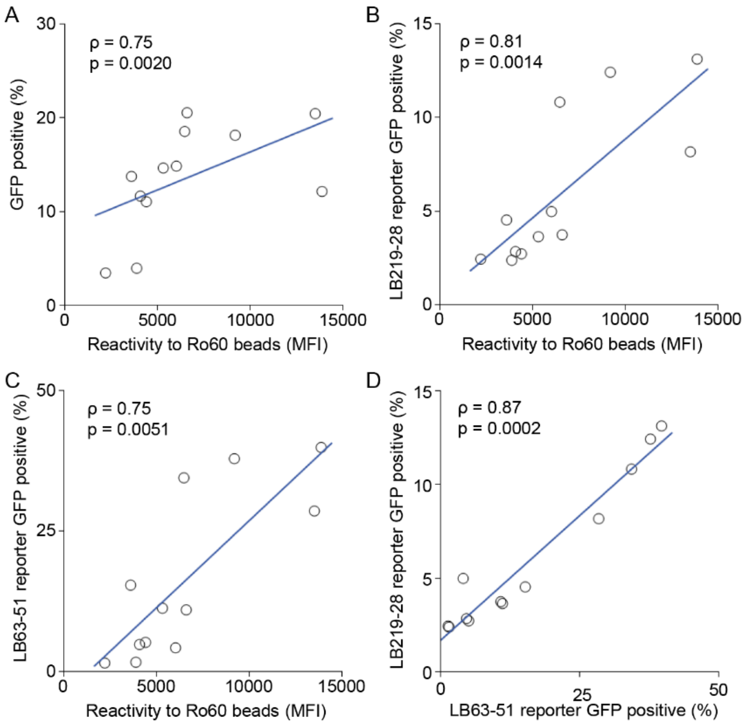

Fig. S5. Reactivity of CD16 reporter and TCR reporter to Ro60-containing immune complexes

(**A**) The correlation between the reactivity of the CD16 reporter and the anti-Ro60 titre of IgG used to form the immune complex is shown. The titre was measured as median fluorescence intensity (MFI) by bead assay. The correlation between the reactivity of (**B**) LB219-28 reporter or (**C**) LB63-51 reporter and the anti-Ro60 titre of IgG used is shown. (**D**) The correlation between the reactivity of LB219-28 and the LB63-51 reporter is shown. Spearman's rank correlation coefficient.

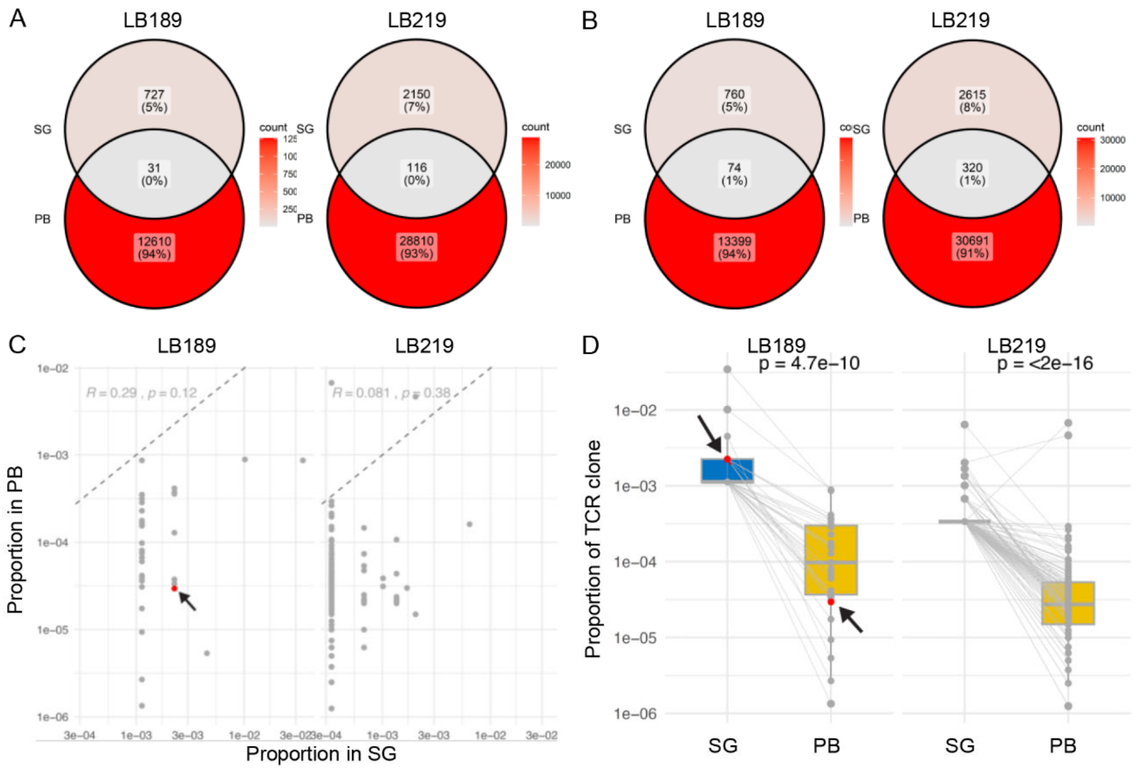

Fig. S6. Comparison of TCR repertoire between salivary glands and peripheral blood

(**A**) The number of shared TCR clonotypes and (**B**) the estimated cell numbers of shared TCR clonotypes between the salivary gland and peripheral blood. (**C**) Correlation of the relative frequencies of clonotypes commonly detected in the salivary gland and peripheral blood. The arrow indicates the Ro60-specific TCR clone LB189-8. Spearman's rank correlation coefficient. (**D**) Comparison of the relative frequencies of clonotypes commonly detected in the salivary gland and peripheral blood. The arrow indicates the Ro60-specific TCR clone LB189-8. A paired Wilcoxon signed-rank test. SG, salivary glands; PB, peripheral blood

This study							Database									
TCR	TRAV	TRAJ	TRA-CDR3	TRBV	TRBJ	TRB-CDR3	complex.id	TRAV	TRAJ	TRA-CDR3	TRBV	TRBJ	TRB-CDR3	MHC A	Epitope species	Epitope gene
LB63-51	TRAV3*01	TRAJ5*01	CAVRNTGRRALTF	TRBV12-4*01	TRBJ2-7*01	CASSYTGAGIHEQYF	11203	TRAV1-2*01	TRAJ5*01	CAVRGTGRRALTF	TRBV20-1*01	TRBJ2-7*01	CSAGGTGIYGSYEQYF	HLA-A*02:01	SARS-CoV-2	Spike
							18503	TRAV1-1*01	TRAJ5*01	CAVRDTGRRALTF	TRBV7-9*01	TRBJ1-1*01	CASSLGDNTEAFF	HLA-A*03:01	CMV	IE1
							23388	TRAV1-1*01	TRAJ5*01	CAVRDTGRRALTF	TRBV7-9*01	TRBJ1-1*01	CASSLGDNTEAFF	HLA-A*03:01	CMV	IE1
							28898	TRAV20*01	TRAJ5*01	CAVRDTGRRALTF	TRBV24-1*01	TRBJ2-2*01	CATSDRSQGVNTGELFF	HLA-A*03:01	CMV	IE1
							32540	TRAV21*01	TRAJ5*01	CAVRRTGRRALTF	TRBV19*01	TRBJ1-4*01	CASSSPVTDPQNEKLFF	HLA-A*03:01	CMV	IE1
							43206	TRAV3*01	TRAJ5*01	CAVRDTGRRALTF	TRBV9*01	TRBJ2-1*01	CASSVPLARGPDEQFF	HLA-B*08:01	HIV-1	Nef
LB189-8	TRAV8-3*01	TRAJ34*01	CAVFLYNTDKLIF	TRBV6-5*01	TRBJ2-5*01	CASSYRRKETQYF	9167	TRAV36/DV7*0	TRAJ34*01	CAVSLYNTDKLIF	TRBV2*01	TRBJ2-7*01	CASSELRYEQYF	HLA-A*02:01	InfluenzaA	M
							20651	TRAV41*01	TRAJ34*01	CAVRLYNTDKLIF	TRBV5-6*01	TRBJ2-7*01	CASSLFHEQYF	HLA-A*03:01	CMV	IE1
							20773	TRAV41*01	TRAJ34*01	CAVRLYNTDKLIF	TRBV5-6*01	TRBJ2-7*01	CASSLFHEQYF	HLA-A*03:01	EBV	EBNA3A
							20782	TRAV3*01	TRAJ34*01	CAVRLYNTDKLIF	TRBV12-4*01	TRBJ2-5*01	CASLLEGAEGETQYF	HLA-A*03:01	CMV	IE1
							27981	TRAV12-2*01	TRAJ34*01	CAVLLYNTDKLIF	TRBV6-5*01	TRBJ1-1*01	CASSYGEGTEAFF	HLA-A*03:01	CMV	IE1
							34578	TRAV39*01	TRAJ34*01	CAVVLYNTDKLIF	TRBV27*01	TRBJ2-5*01	CASMGNFQETQYF	HLA-A*03:01	CMV	IE1
							40566	TRAV21*01	TRAJ34*01	CAVPLYNTDKLIF	TRBV9*01	TRBJ2-3*01	CASSVDLGTSGGSDTQYF	HLA-B*08:01	HomoSapiens	G6PC2
LB219-28	TRAV35*02	TRAJ40*01	CAGLLTSGTYKYIF	TRBV5-1*01	TRBJ2-5*01	CASSSKQGLGKTQYF	9715	TRAV35*01	TRAJ40*01	CAGLPTSGTYKYIF	TRBV19*01	TRBJ2-2*01	CASSIFSVTELFF	HLA-A*02:01	InfluenzaA	M
							36429	TRAV27*01	TRAJ40*01	CAGLPTSGTYKYIF	TRBV5-1*01	TRBJ1-1*01	CASSEGTGAGAEAFF	HLA-A*11:01	EBV	EBNA3B
LB219-10-2	TRAV26-1*01	TRAJ44*01	CIVRVFTGTASKLTF	TRBV6-6*01	TRBJ2-7*01	CASSSLGYEQYF	1192	TRAV19*01	TRAJ39*01	CALNPQNAGNMLTF	TRBV12-3*01	TRBJ2-7*01	CASSLLGYEQYF	HLA-A*02:01	SARS-CoV-2	Nucleocapsid
							7458	TRAV4*01	TRAJ8*01	CLVRGTGFQKLVF	TRBV28*01	TRBJ2-7*01	CASSSQGYEQYF	HLA-DRA*01:01	InfluenzaA	HA
							11152	TRAV8-4*01	TRAJ3*01	CAVPTYSSASKIIF	TRBV7-2*01	TRBJ2-7*01	CASSSGGYEQYF	HLA-B*07:02	SARS-CoV-2	Nucleocapsid
							27594	TRAV8-3*01	TRAJ47*01	CAVGAGGKLVF	TRBV7-2*01	TRBJ2-7*01	CASSSAGYEQYF	HLA-A*11:01	EBV	EBNA3B
							27812	TRAV12-2*01	TRAJ49*01	CAVNRNSNQFYF	TRBV4-1*01	TRBJ2-7*01	CASSSGGYEQYF	HLA-A*03:01	CMV	IE1
							32838	TRAV38-2/DV8	TRAJ20*01	CASLSSNDYKLSF	TRBV12-4*01	TRBJ2-7*01	CASSSDGYEQYF	HLA-A*03:01	CMV	IE1
JCI-7E	TRAV8-4*01	TRAJ17*01	CAVSESGAAGNKLTF	TRBV7-9*01	TRBJ2-7*01	CASSPRDSYEQYF	6152	TRAV19*01	TRAJ45*01	CALSEASSGGGADGLTF	TRBV27*01	TRBJ2-7*01	CASSIRDSYEQYF	HLA-E*01:03	Mtb	Rv1734c
							10717	TRAV8-6*01	TRAJ37*01	CAVSDDEGKLIF	TRBV19*01	TRBJ2-7*01	CASSPRSSYEQYF	HLA-A*02:01	InfluenzaA	M
							21872	TRAV38-1*01	TRAJ52*01	CAFMRGRGKLTF	TRBV7-9*01	TRBJ2-7*01	CASSFRDSYEQYF	HLA-A*11:01	EBV	EBNA3B
							22347	TRAV38-2/DV8	TRAJ34*01	CAYRSEGGTDKLIF	TRBV7-9*01	TRBJ2-7*01	CASSFRDSYEQYF	HLA-B*08:01	EBV	BZLF1
							22353	TRAV13-2*01	TRAJ45*01	CADSGGGADGLTF	TRBV19*01	TRBJ2-7*01	CASSPRSSYEQYF	HLA-B*08:01	EBV	BZLF1
							22847	TRAV38-1*01	TRAJ52*01	CAFMRGRGKLTF	TRBV7-9*01	TRBJ2-7*01	CASSFRDSYEQYF	HLA-B*08:01	EBV	BZLF1
							25160	TRAV12-2*01	TRAJ42*01	CAVNEGGGSQGNLIF	TRBV19*01	TRBJ2-7*01	CASSPRSSYEQYF	HLA-A*02:01	InfluenzaA	M
							25589	TRAV38-1*01	TRAJ52*01	CAFMRGRGKLTF	TRBV7-9*01	TRBJ2-7*01	CASSFRDSYEQYF	HLA-A*03:01	CMV	IE1
							33922	TRAV25*01	TRAJ11*01	CAVDSGYSTLTF	TRBV4-1*01	TRBJ2-7*01	CASSPPDSYEQYF	HLA-A*03:01	CMV	IE1
							37022	TRAV8-4*01	TRAJ11*01	CAVSDQGYSTLTF	TRBV5-1*01	TRBJ2-7*01	CASSPTDSYEQYF	HLA-A*11:01	EBV	EBNA4
							45424	TRAV19*01	TRAJ45*01	CALSEASSGGGADGLTF	TRBV27*01	TRBJ2-7*01	CASSIRDSYEQYF	HLA-E*01:03	Mtb	Rv1734c
JCI-8E	TRAV20*01	TRAJ13*01	CAVQAIPGGYQKVTF	TRBV24-1*01	TRBJ2-2*01	CATGEQNTGELFF	11002	TRAV12-1*01	TRAJ34*01	CVVNKDDKLIF	TRBV12-3*01	TRBJ2-2*01	CALGEQNTGELFF	HLA-A*02:01	SARS-CoV-2	Spike
							38221	TRAV29/DV5*0	TRAJ52*01	CAASRPSGGTSYGKLTF	TRBV10-1*01	TRBJ2-2*01	CATGESNTGELFF	HLA-A*02:01	SARS-CoV-2	Spike

Fig. S7. Sequence similarity analysis

TCRs on the database with CDR3 regions either identical or with one amino acid difference from the Ro60-specific TCR are listed. Red text indicates homologous to the Ro60-specific TCR.

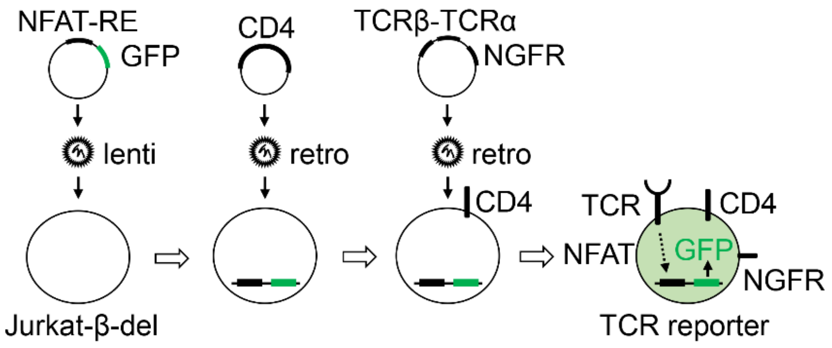

Fig. S8. Generation of TCR reporter cells

The stepwise method for generating TCR reporters is shown. GFP, green fluorescent protein; NFAT-RE, NFAT response element; NGFR-ECD, extracellular domain of nerve growth factor receptor.

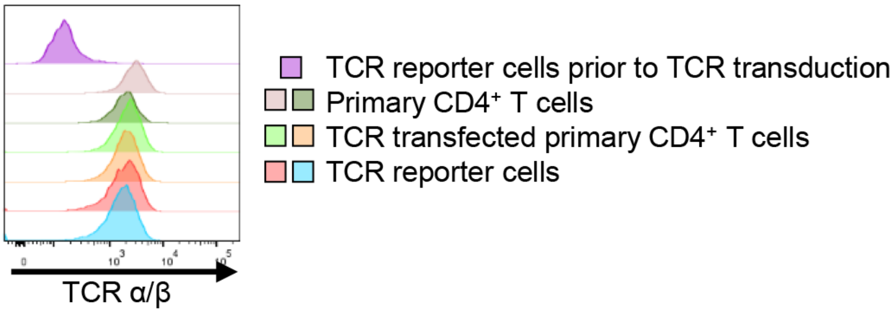

Fig. S9. Comparison of cell-surface TCR expression

Cell-surface TCR levels on representative primary $CD4^+$ T cells, TCR-transfected primary $CD4^+$ T cells, and TCR-reporter cells were analyzed by FACS and compared with those of TCR-reporter cells prior to TCR transduction.

Table S1. Clinical information of the patients

Patients ID	LB63	LB65	LB183	LB189	LB214	LB215	LB216	LB219	LB220	LB221
Age	49	49	53	44	66	52	32	76	83	80
Sex	Female	Female	Female	Female	Female	Male	Female	Female	Female	Female
Criteria for SjD	Fulfilled	Fulfilled	Fulfilled	Fulfilled	Fulfilled	Fulfilled	Fulfilled	Fulfilled	Fulfilled	Fulfilled
Other autoimmune disease	−	−	−	RA	−	RA	SLE	−	−	−
Greenspan grade	4	4	3	4	4	4	4	4	1	4
Serum anti-Ro60 Ab	+	+	+	+	+	+	+	+	+	+
Serum anti-Ro52 Ab	−	−	−	+	−	+	−	+	+	−
Serum anti-SSB Ab	−	−	−	−	−	+	−	−	−	−
Serum RF	−	−	−	+	−	+	−	+	ND	+
Treatment	None	None	None	None	None	None	None	None	None	None
HLA typing										
HLA-DRB1	04:06, 15:01	04:01, 15:01	08:03, 08:09	14:06, 15:01	08:03, 14:05	08:09, 15:02	08:03, 0803	14:06, 15:01	ND	09:01, 09:01
HLA-DRB3/4/5	4*01:03, 5*01:01	4*01:02, 5*01:01	−	3*02:02, 5*01:01	3*02:02	5*01:02	−	3*02:02, 5*01:01	ND	4*01:03, 4*01:03
HLA-DQA1	01:02, 03:01	01:02, 03:03	01:03, 04:01	01:02, 05:03	01:04, 06:01	01:03, 04:01	01:03, 01:03	01:02, 05:03	ND	03:02, 03:02
HLA-DQB1	03:02, 06:02	03:01, 06:02	04:02, 06:01	03:01, 06:02	03:01, 05:03	04:02, 06:01	06:01, 06:01	03:01, 06:02	ND	03:03, 03:03
HLA-DPA1	01:03, 02:02	01:03, 02:02	02:02, 02:02	01:03, 02:02	01:03, 02:02	02:01, 02:02	01:03, 02:02	01:03, 02:02	ND	01:03, 0201
HLA-DPB1	02:01, 03:01	05:01, 36:01	02:02, 05:01	02:01, 05:01	02:01, 05:01	05:01, 09:01	05:01, 05:01	02:01, 05:01	ND	01:03, 0501
Number of tested TCRs	25	20	6	38	−	14	22	48	−	3
TCR cloning methods	Sorting	Sorting	Chromiu m	Chromiu m	Chromiu m	Chromiu m	Chromiu m	Chromiu m	Chromiu m	Chromiu m
Tissue anti-Ro60 Ab	ND	ND	+	+	ND	+	+	+	ND	+
Methods for validating tissue antibody specificity			sorting IS	sorting IS		IS	sorting	IS		IS

RA, rheumatoid arthritis; SLE, systemic lupus erythematosus; Ab, antibody; RF, rheumatoid factor; ND, not determined; IS, immunostaining.

Table S2. HLA haplotypes of HLA-matched PBMC, monocytes, and monocyte-derived DCs

required HLA	HLA-DRB1*14:06	HLA-DBR1*15:01 and HLA-DQA1*01:02 HLA-DQB1*06:02	HLA-DPA1*02:02 HLA-DPB1*05:05
Figure	4B,C	4D,E,G,H,I,J	4D,F
ID	LB189	HC1	HC2
Sex	Female	Male	Male
HLA typing			
HLA-DRB1	14:06, 15:01	15:01, 15:01	04:05, 04:05
HLA-DRB3/4/5	3*02:02, 5*01:01	5*01:01, 5*01:01	4*01:03, 4*01:03
HLA-DQA1	01:02, 05:03	01:02, 01:02	03:03, 03:03
HLA-DQB1	03:01, 06:02	06:02, 06:02	04:01, 04:01
HLA-DPA1	01:03, 02:02	02:02, 02:02	02:02, 02:02
HLA-DPB1	02:01, 05:01	02:01, 05:01	05:01, 05:01

Table S3. Antibodies

Antibody	Source	Identifier
APC/Cyanine7 anti-human CD3 antibody	BioLegend	300426
PE/Cyanine7 anti-human CD4 antibody	BioLegend	344612
Brilliant Violet 510 anti-human CD8a antibody	BioLegend	301048
PE/Cyanine7 anti-human CD16 antibody	BioLegend	302016
Brilliant Violet 421 anti-human CD19 antibody	BioLegend	302234
FITC anti-human CD38 antibody	BioLegend	303504
APC anti-human CD45 antibody	BioLegend	304012
PE anti-human CD326 (EpCAM) antibody	BioLegend	324205
PE anti-human CD271 (NGFR) antibodies	BioLegend	345106
APC anti-human TCRα/β antibody	BioLegend	306718
PE mouse anti-human CD154	BD Biosciences	555700
Brilliant Violet 510 anti-human IFNγ antibody	BioLegend	502544
Anti-CD138 antibody	BioLegend	356502
Ultra-LEAF purified anti-human HLA-DR	BioLegend	307666
Ultra-LEAF purified anti-human HLA-A, B, C	BioLegend	311428
Ultra-LEAF purified anti-human CD3 antibody	BioLegend	300437
Ultra-LEAF purified anti-human CD28 antibody	BioLegend	302934
Ultra-LEAF™ Purified Mouse IgG2a, κ Isotype Ctrl Antibody	BioLegend	401508
Ultra-LEAF™ Purified Mouse IgG3, κ isotype Ctrl Antibody	BioLegend	401327
Anti-HLA-DQ, human	Leinco Technologies	H262
Anti-HLA-DP, human	Leinco Technologies	H260
Alexa Fluor® 647 F(ab′)$_2$ fragment Goat anti-human IgG, Fcγ fragment specific	The Jackson Laboratory	109-606-098
Goat anti-mouse IgG (H+L) highly cross-adsorbed secondary antibody, Alexa Fluor Plus 594	Thermo Fisher Scientific	A32742

Data S1. (separate file)

PCR primer sequences.
